# PMA1-containing extracellular vesicles of *Candida albicans* triggers immune responses and colitis progression

**DOI:** 10.1080/19490976.2025.2455508

**Published:** 2025-01-31

**Authors:** Zhen Xu, Shuping Qiao, Zelin Wang, Chen Peng, Yayi Hou, Baorui Liu, Guochun Cao, Tingting Wang

**Affiliations:** a Department of Oncology, Nanjing Drum Tower Hospital, State Key Laboratory of Pharmaceutical Biotechnology, Affiliated Hospital of Medical School, Nanjing University, Nanjing, China; b The Comprehensive Cancer Centre of Drum Tower Hospital, Medical School of Nanjing University & Clinical Cancer Institute of Nanjing University, Nanjing, China; c Jiangsu Key Laboratory of Molecular Medicine, Division of Immunology, Medical School, Nanjing University, Nanjing, China; d Department of Medical Oncology, Jiangsu Cancer Hospital & Jiangsu Institute of Cancer Research & The Affiliated Cancer Hospital of Nanjing Medical University, Nanjing, China

**Keywords:** *C. albicans*, extracellular vesicles, PMA1, dendritic cell, colitis

## Abstract

*Candida albicans* (*C. albicans*) exhibits aberrant changes in patients with colitis, and it has been reported to dominate the colonic mucosal immune response. Here, we found that PMA1 expression was significantly increased in *C. albicans* from patients with IBD compared to that in healthy controls. A Crispr-Cas9-based fungal strain editing system was then used to knock out PMA1 expression in *C. albicans*. Compared to *WT-C.a*, *ΔPMA1-C.a* could not aggravate colitis. Proteomic analysis showed that PMA1 was transported by extracellular vesicles (EVs) of *C. albicans*. PMA1-containing EVs aggravated colitis, modulated the migration of cDC2 from the lamina propria to mesenteric lymph nodes, and induced TH17 cell differentiation. Moreover, the adaptor protein CARD9 was critical in PMA1-containing EV-induced colitis, and CARD9-deficient DCs did not induce TH17 cell differentiation or IL-17A production. Mechanically, CARD9 combines with the glycolytic protein GAPDH (aa2–146 domain) through its CARD region. CARD9 deficiency led to decreased enzyme activity of GAPDH and decreased glycolysis of DCs. These findings indicate that PMA1 is a potential virulence factor responsible for the pathogenesis of *C. albicans* colitis.

## 1. Introduction

Inflammatory bowel disease (IBD), which can be divided into Crohn’s disease (CD) and ulcerative colitis (UC), is characterized by chronic, relapsing inflammation of the gastrointestinal tract. Previous studies have indicated that IBD is a polygenic and polymicrobial disease.^1^

Since the gut microbe is considered an important organ of the human body, an increasing number of studies have linked gut microbes to disease. Several types of microbiota are highly related to IBD, including *Faecalibacterium prausnitzii*,^2^ *Escherichia coli*,^3^ and *Fusobacterium*.^4^ The interaction between the host and microbe influences the development and progression of IBD.^5,6^ In addition to bacteria, the sequencing results of intestinal flora revealed significant fungal alteration in feces and mucosa samples in IBD. Although fungi constitute approximately 0.1% of all microorganisms in the gut, they have been proven to play a critical role in IBD pathogenesis.^7^ Interestingly, pilot studies of the supplementing fungal probiotics *Saccharomycopsis fibuligera*, *Saccharomyces boulardii*, and *Saccharomyces cerevisiae* showed therapeutic effects against IBD.^8^ Recently, a rich genetic diversity of opportunistic *Candida albicans* strains was shown to dominate the colonic mucosa of patients with IBD.^7^ It was reported that *C. albicans* evolved hyphal-specific factors to better compete with bacterial species in the intestinal niche and promote its gut commensalism.^9^ The ability of *C. albicans* to infect hosts is supported by various virulence factors, including polysaccharides^10^ and the secretion of specific toxins such as candidalysin.^11–13^ However, the characteristic changes of *C. albicans* in the gastrointestinal tract of patients with IBD remain unclear.

Extracellular vesicles (EVs) are evolutionarily conserved nanomembrane vesicles secreted by both prokaryotic and eukaryotic cells.^14^ In recent years, EVs secreted by microorganisms have gained considerable attention for their roles in cellular communication and may have value as biomarkers for diagnosis and prediction.^15,16^ EVs are released from the surface of fungi and contain a suite of molecular cargo, such as soluble proteins, sRNA, or virulence factors, which they deliver to target cells.^17,18^ Models of fungal EVs stimulating host cells *in vitro* and *in vivo* clearly indicate that these compartments are immunologically active and may represent a key pathogenic step during fungal infection.^19–21^

Intestinal homeostasis is tightly controlled by immunomodulatory mechanisms established by microorganisms and their microbial products that interact with immune cells.^22^ Innate cells (e.g., dendritic cells [DCs], macrophages) are the initial targets of pathogenic microorganisms and their microbial products, which subsequently influence the regulation of intestinal immunity.^1,23,24^ In the intestinal mucosa, various subpopulations of DCs are located diffusely throughout the intestinal lamina propria (LP), within gut-associated lymphoid tissues, including mesenteric lymph nodes (MLN) and Peyer’s patches.^25,26^ LP DCs can take up antigens in the intestine and migrate through the lymph to draining MLNs, where they can present antigens to T cells, promote effector T-cell differentiation, and promote cytokine secretion.^25,27^ Recent evidence has demonstrated the functional diversity of each subset.^28^ It is important to describe the regulatory factors of DC subset development and functionality to provide novel therapeutic targets in IBD. When this balance is disrupted by microbial signaling, highly activated innate cells trigger subsets of pathogenic T cells that infiltrate the gut (e.g., TH17) and even regulatory T cells (Tregs) with proinflammatory characteristics,^29,30^ which eventually leads to tissue destruction and intestinal disease progression. However, the pathogenic agents of *C. albicans* are unclear, and their molecular mechanisms that influence the immune response have not been elucidated.

Here, we identified PMA1 as a pathogenic agent of *C. albicans*. PMA1, which is carried by EVs and activates DCs, promotes cytokine secretion and migration of the cDC2 subset in the gut, thereby promoting TH17 differentiation and progression of colitis.

## 2. Results

### 2.1. PMA1 is a pathogenic factor of *C.* albicans that aggravates colitis

**Figure 1.**
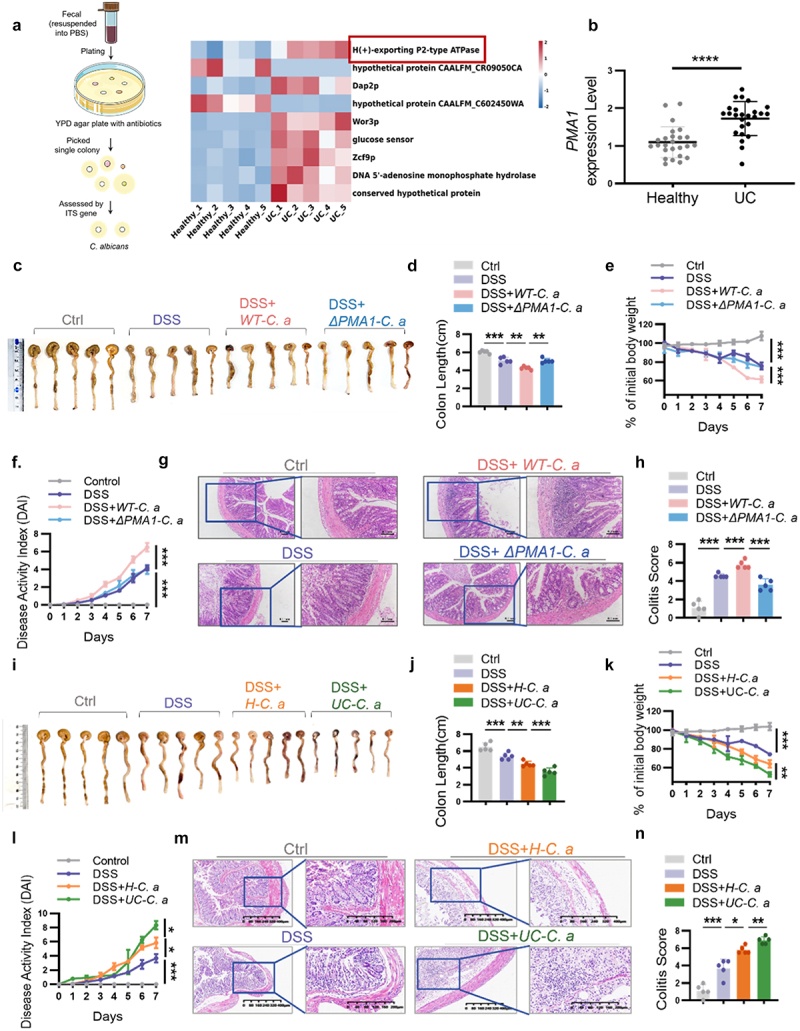
PMA1 of *C. albicans* is a pathogenic factor that aggravates colitis.

To study the characteristic differences of *C. albicans* in the gut between healthy controls and patients with IBD, fecal samples were collected from non-IBD controls (healthy, *n* = 5) and patients with UC (UC, *n* = 5). *C. albicans* colonies in fecal samples were picked using specific YPD agar plates with antibiotics and were confirmed by qPCR (Figure 1(a), left). RNA-seq was used to identify the gene expression of *C. albicans* in healthy people and patients with UC. As shown in Figure 1(a) (right), PMA1 (H (+) – exporting P2-type ATPase) was significantly increased in *C. albicans* isolated from patients with UC compared to healthy controls. To confirm this result, we selected five *C. albicans* colonies from each patient and measured the expression of PMA1 using qPCR. The results indicated that PMA1 expression was higher in *C. albicans* isolated from patients with UC than those isolated from healthy controls (Figure 1(b)).

(a) Flow chart of *C. albicans* isolation from fecal samples (left). Differentially expressed genes in *C. albicans* from healthy people (*n* = 5) or patients with UC (*n* = 5) were detected by RNA sequencing. Heatmap of the differentially expressed genes in *C. albicans*(fold change > 2, *p* < 0.05). Each dot represents an individual human participant (right). (b) Stool samples were collected from healthy individuals (*n* = 5) and patients with UC (*n* = 5). Five *C. albicans* colonies were picked from each person’s stool. The mRNA expression of *PMA1* in *C. albicans* was detected using qPCR. Each dot represents one colony of *C. albicans*. (c – h) Mice were orally treated with 1 × 10^8^ *WT-C.a* or *ΔPMA1-C. a* (*n* = 5 of each group) during DSS-induced colitis. The colon length, weight change, and DAI score were measured, representative H&E images are shown (scale bar: 0.1 mm), and colitis scores were calculated. (i – n) Mice were orally treated with 1 × 10^8^ *C. albicans* isolates from healthy people (*H-C.a*) or patients with UC (*UC-C.a*) (*n* = 5 of each group) during DSS-induced colitis. The colon length, weight change, and DAI score were measured, representative H&E images are shown (scale bar: 400 μm), and colitis scores were calculated. For (c)–(j), each dot represents an individual mouse. Data with error bars are represented as the mean ± SD. *P < 0.05, **P < 0.01, ***P < 0.001, ****P < 0.0001 as determined by unpaired Student’s t test.

To investigate the functional role of PMA1 in *C. albicans*, we constructed a ΔPMA1-*C. albicans* (*ΔPMA1-C.a*) mutant strain using a CRISPR-based technique in WT-*C. albicans* (*WT-C.a*). The construction process of the *ΔPMA1-C.a* strain and the confirmation of PMA1 knockout in *C. albicans* are shown in Figure S1A. We then examined the growth curves of the two species of *C. albicans* and found that PMA1 knockout did not affect the growth of *C. albicans* (Figure S1B). Compared to *ΔPMA1-C.a*, *WT-C.a* contributed to severe intestinal inflammation in DSS-induced colitis, which manifested as a shortened colon, decreased body weight, increased DAI scores, increased monocyte infiltration, and increased colitis score (Figure 1(c–h)). To confirm the endogenous role of PMA1 during the pathogenesis of *C. albicans*, we treated mice with *C. albicans* isolated from healthy controls (*H-C.a*) and patients with UC (*UC-C.a*). As shown in Figure 1(i–n), colitis mice treated with *UC-C.a* exhibited a shortened colon, decreased body weight, increased DAI scores, increased mucosal erosion, increased inflammatory cell infiltration, and increased colitis score compared to those treated with *H-C.a*.

We also investigated the role of *C. albicans* in spontaneous colitis by inoculating mice with *WT-C.a*, *ΔPMA1-C.a*, *H-C.a*, and *UC-C.a*. without DSS treatment. As shown in Figure S1C – D, these different types of *C. albicans*, regardless of PMA1 expression, did not cause spontaneous colitis. Our results suggest that *C. albicans* possesses a specific pathogenic factor, PMA1, that promotes the progression of colitis.

### 2.2. PMA1 in *C.* *albicans* activates DCs and triggers TH17 differentiation

**Figure 2.**
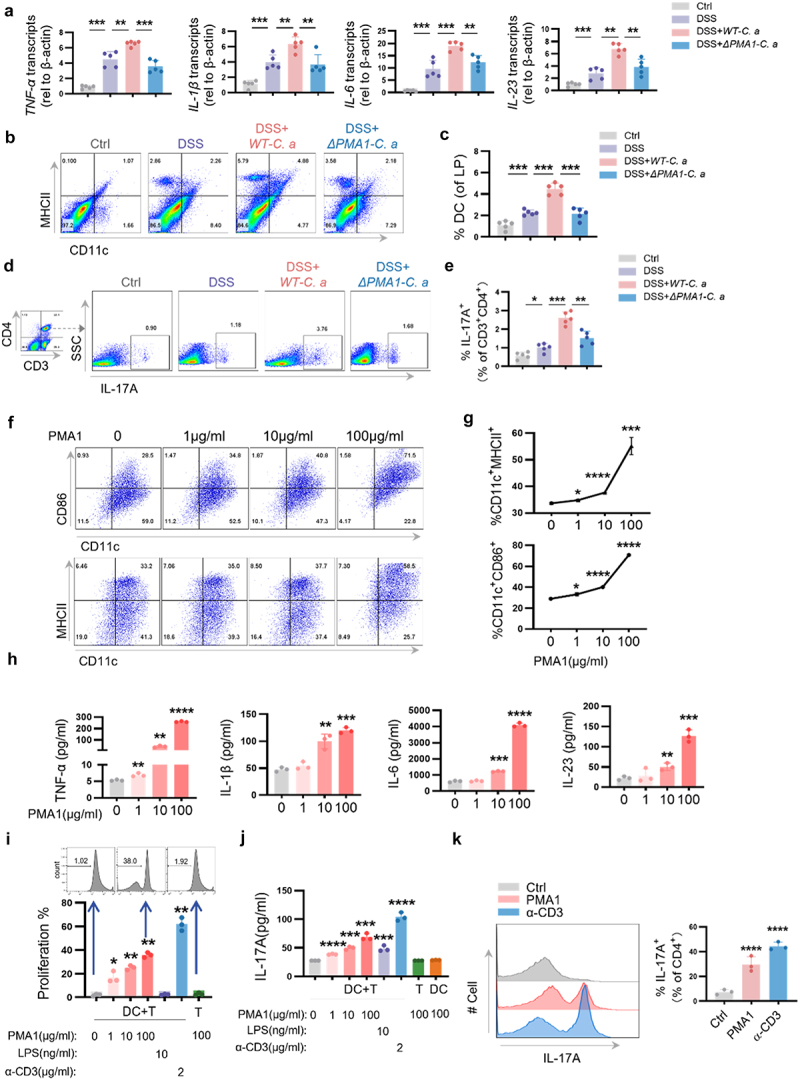
PMA1 of *C. albicans* activates DCs and triggers differentiation of TH17.

To explore the immunomodulatory properties of PMA1, we analyzed the expression of inflammatory factors and the composition of immune cells in the above mouse model. As shown in Figure 2(a), *ΔPMA1-C.a* treatment led to decreased mRNA expression of *TNF-α*, *IL-6*, *IL-1β*, and *IL-23* in the colon compared to *WT-C.a* treatment. Similar results were found in colons from the *H-C.a* group compared to the *UC-C.a* group (Figure S2A). We also found lower proportions of DCs (CD11c^+^ MHC-II^+^) and TH17 cells (CD3^+^CD4^+^IL-17A^+^) in the LP of colons in *ΔPMA1-C.a* treated mice than those in *WT-C.a* treated mice (Figure 2(b–e)). However, the proportions of macrophages, neutrophils, TH1, TH2, and Treg were not changed in the *ΔPMA1-C.a* treated group compared to the *WT-C.a* treated group (Figure S2B). Similar results were found in colons from the *H-C.a* group compared to those from the *UC-C.a* group (Figure S2C – D).

(a – e) Mice were treated as described in Figure 1c. Colon tissues were collected. Each dot represents an individual mouse. (a) The mRNA expression of *TNF-α*, *IL-1β*, *IL-6*,and *IL-23*in colons was detected using qPCR. (b – c) Proportion of DCs (CD11c^+^ MHCII^+^) in the LP was detected by flow cytometry. (D – E) Proportion of TH17 (CD3^+^CD4^+^IL-17A^+^) in the LP was detected by flow cytometry. (f – h) BMDCs were separated from C57BL/6 mice and treated with purified PMA1protein for 24 h. (f – g) Expression of CD86 and MHCII was detected by flow cytometry. (H) Production of TNF-α, IL-1β, IL-6, and IL-23 was assessed by ELISA. (i – k) BMDCs were treated with purified PMA1 protein for 24 h, before co-culturing with CD4^+^ T cells. (i) Proliferation of CD4^+^T cells was detected by flow cytometry. (j) Production of IL-17A in cellular supernatant was assessed by ELISA. (K) Proportion of IL-17A^+^CD4^+^ T cells was detected by flow cytometry. Data with error bars are represented as the mean ± SD. Each panel is a representative experiment of at least three independent biological replicates. *P < 0.05, **P < 0.01, ***P < 0.001, ****P < 0.0001 as determined by unpaired Student’s t test.

We then explored the immunomodulatory properties of PMA1 *in vitro*. To this end, bone marrow-derived dendritic cells (BMDCs) were cultured and treated with purified PMA1 protein. As shown in Figure 2(f–g), PMA1 induced the maturation of DCs, presenting as increased expression of CD86 and MHCII among CD11c^+^ cells, which was dose dependent. The secreted proinflammatory factors of BMDCs were also assessed. The protein levels of TNF-α, IL-1β, IL-6, and IL-23 were significantly increased after PMA1 stimulation (Figure 2(h)). Furthermore, naïve splenic CD4^+^ T cells were isolated and co-cultured with BMDCs. PMA1-treated BMDCs led to increased proliferation of CD4^+^ T cells, increased secretion of IL-17A, and an increased proportion of TH17 cells (Figure 2(i–k)). However, PMA1-treated BMDCs had no significant effect on the differentiation of TH1, TH2, or Treg cells (Figure S2E – F). Moreover, the effect of PMA1 on CD4^+^ T cells required the participation of DCs, as PMA1 alone could not stimulate T cell activation (Figure 2(i–k)). Together, these results showed that PMA1 can stimulate the maturation of DCs and the differentiation of TH17 *in vivo* and *in vitro*.

### 2.3. PMA1 is packaged into EVs to aggravate colitis

**Figure 3.**
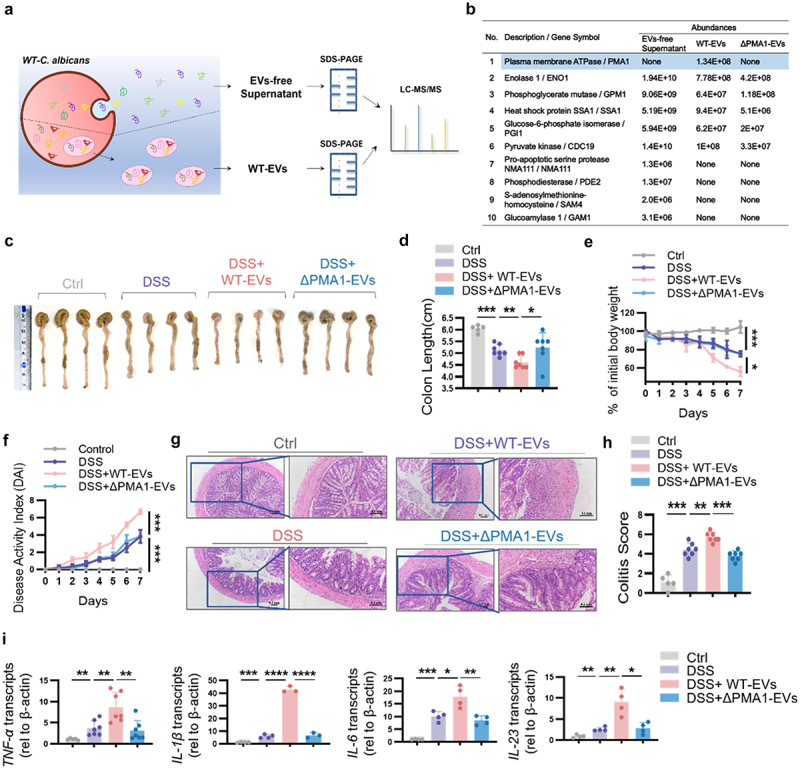
PMA1-containing EVs aggravate colitis.

To investigate the mechanism by which *C. albicans* secretes PMA1, the proteome composition of the EVs and EVs-free supernatant was analyzed in parallel according to the scheme shown in Figure 3(a). As shown in Figure 3(b), PMA1 is present only in EVs and cannot be secreted in an autocrine manner. Therefore, EVs from *WT-C.a* and *ΔPMA1-C.a* were separated. Scanning electron microscopy (SEM) and transmission electron microscopy (TEM) revealed that the putative EVs protrusion, particle size, and abundance did not differ significantly between *WT-C.a*-derived EVs (WT-EVs) and *ΔPMA1-C.a*-derived EVs (ΔPMA1-EVs, Figure S3A). Furthermore, deficient expression of PMA1 was confirmed in *ΔPMA1-C.a*-derived EVs (Figure S3B). Together, these findings revealed that *C. albicans* packaged PMA1 into EVs, potentially representing a mechanism by which the mammalian host delivers immunomodulatory signals.

(a) Cell-free culture supernatants from *C. albicans* were separated into EVs and EVs-free supernatant, and then analyzed by LC−MS/MS. (b) The protein abundance in the EVs-free supernatant sample was compared to the WT-EV sample and ΔPMA1-EVs. (c–i) Mice were orally treated with WT-EVs or ΔPMA1- EVs (*n* = 10 of each group) during DSS-induced colitis. (c–h) The colon length, weight change, and DAI score were measured, representative H&E images are shown (scale bar: 0.1 mm), and colitis scores were calculated. (i) The mRNA expression of *TNF-α*, *IL-1β*, *IL-6*, and *IL-23* in colon tissues was detected using qPCR. For (d)–(g), each dot represents an individual mouse. Data with error bars are represented as the mean ± SD. *P < 0.05, **P < 0.01, ***P < 0.001, ****P < 0.0001 as determined by unpaired Student’s t test.

To investigate whether PMA1-containing EVs have pathogenic activity, we orally treated mice with WT-EVs and ΔPMA1-EVs. Measurements of the colon length showed colon shortening in mice with colitis, which deteriorated after the oral administration of PMA1-containing vesicles (Figure 3(c–d)). The results in Figure 3(e–f) showed that the weight loss and DAI scores were increased in the WT-EVs group compared to those in the colitis model. Upon histological analysis of colonic tissues, we observed significant infiltration of inflammatory cells in the colons of the WT-EVs group compared to that from the ΔPMA1-EVs group (Figure 3(e–f)). The mRNA expression of *TNF-α*, *IL-1β*, *IL-6*, and *IL-23* was also elevated in the colonic tissues of the DSS group. Compared to ΔPMA1-EVs treatment, WT-EVs treatment enhanced the expression of these proinflammatory cytokines in colon tissue (Figure 3(g)). Consistent with the fungal gavage experiments, *C. albicans* EVs did not cause spontaneous colitis without DSS treatment (Figure S3C – D). These results suggest that PMA1 packaging into EVs increases the pathological and immunological manifestations of colitis.

### 2.4. PMA1-containing EVs regulate cDC2 migration and immune responses during colitis

**Figure 4.**
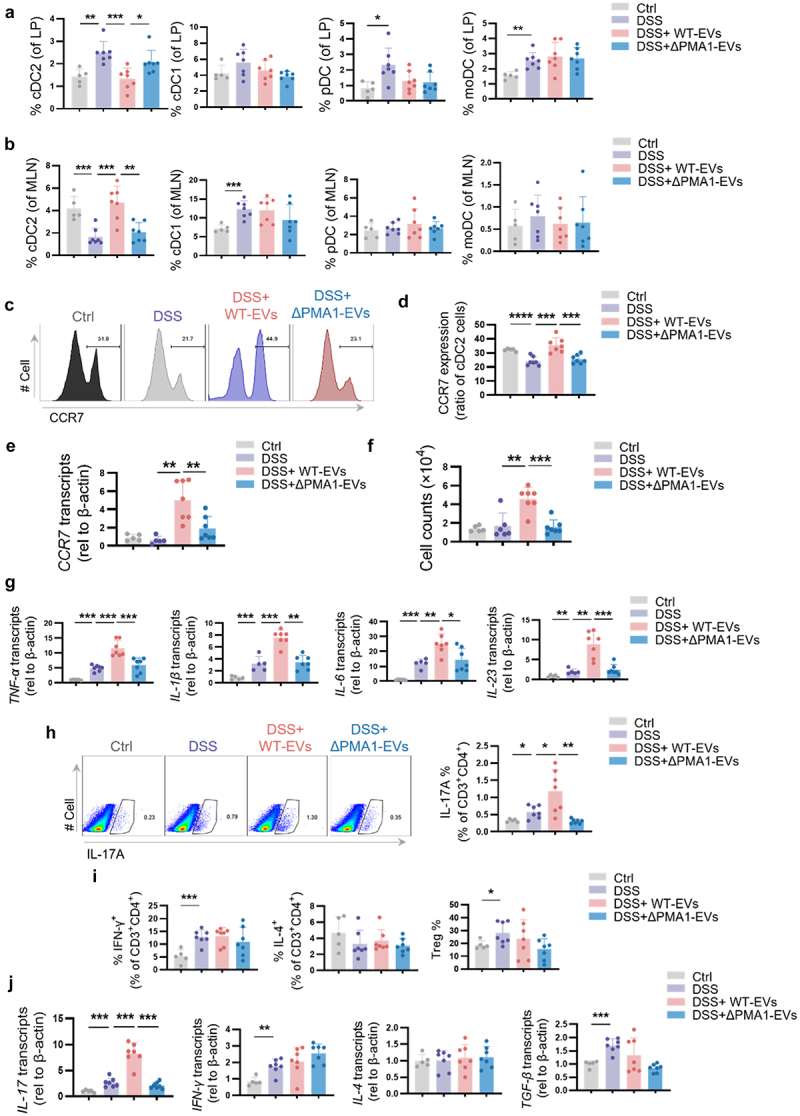
PMA1-containing EVs regulate cDC2 migration and the immune response during colitis.

We next studied the immunomodulatory properties of PMA1-containing EVs. The intestinal mucosa contains a variety of subpopulations of DCs, including conventional DCs (cDCs),^28,31^ plasmacytoid DCs (pDCs),^32^ and monocyte-derived DCs (moDCs).^33,34^ Therefore, flow cytometry was used to identify these DC subsets in the LP and MLN in mice with colitis (Figure S4A – B). Compared to ΔPMA1-EV treated mice, WT-EVs treatment led to a reduced proportion of the cDC2 subset (Lin^−^CD11c^+^MHCII^+^XCR1^−^CD11b^+^ SIRPα^+^) in the LP (Figure 4(a)). Interestingly, the proportion of cDC2 in the MLN was strongly increased upon WT-EVs treatment (Figure 4(b)). However, treatment with EVs had no significant effect on the amounts of cDC1, moDC, and pDC in the LP and MLN (Figure 4(a–b)).

(a – j) Mice were treated as described in Figure 3c. Each dot represents an individual mouse. (a) The percentage of the cDC2 subset was detected by flow cytometry in the LP. (b) The percentage of the cDC2 subset was detected by flow cytometry in the MLN. (c – d) The expression of CCR7 on cDC2 was detected by flow cytometry in the LP. (e – g) The cDC2 subset was separated from the LP by flow sorting. (e) The mRNA expression of *CCR7* was detected by qRCR. (f) cDC2 was cultured in an 8-μm Transwell chamber for 24 h, the number of cells that migrated below the chamber was detected by flow cytometry. (g) The mRNA expression of *TNF-α*, *IL-1β*, *IL-6*, and *IL-23* was detected by qRCR. (h) The proportion of TH17 cells in the LP was detected by flow cytometry. (h) The percentage of TH1, TH2, and Treg cells in the LP was detected by flow cytometry. (j) CD4^+^ T cells were isolated from the mouse MLN by magnetic cell sorting, and the mRNA expression of *IL-17*, *IFN-γ*, *IL-4*,and *TGF-β*was detected using qPCR. Data with error bars are represented as the mean ± SD. * *P* < 0.05, ** *P* < 0.01, *** *P* < 0.001 as determined by unpaired Student’s t test.

The ability of DCs to migrate is critical for initiating protective pro-inflammatory and tolerogenic immune responses.^35^ The opposite changes in cDC2 levels in the MLN and LP prompted us to investigate the migration of cDC2 in the gut. CCR7 chemokine receptor stimulation induces the migration of DCs toward lymph nodes.^35^ We examined CCR7 expression in cDC2 in the LP of colitis mice. As shown in Figure 4(c–d), the protein level of CCR7 was significantly upregulated upon WT-EV treatment, while ΔPMA1-EVs treatment had no significant effect on CCR7 levels. Furthermore, primary cDC2 were separated from the LP by flow sorting. The mRNA expression of CCR7 in primary cDC2 was induced after treatment with WT-EVs compared to that in the DSS group. Meanwhile, ΔPMA1-EVs treatment could not up-regulate CCR7 expression (Figure 4(e)). Next, a cell migration experiment was developed using a Transwell chamber plate. cDC2 separated from mice with colitis treated with WT-EVs showed greater migration ability than those treated with ΔPMA1-EVs (Figure 4(f)). We also detected the expression of cDC2. As shown in Figure 4(g), the expression of *TNF-α*, *IL-1β*, *IL-6*, and IL-23 were significantly induced by WT-EVs, but not ΔPMA1-EVs (Figure 4(g)). Then, we examined the differentiation of CD4^+^ T cells in the LP of mice with colitis by flow cytometry and found that the number of TH17 cells was significantly upregulated by WT-EVs stimulation but downregulated by ΔPMA1-EVs stimulation (Figure 4(h)). However, EVs treatment had no significant effect on the proportions of TH1, TH2, and Treg cells (Figure 4(i)). In addition, we isolated CD4^+^ T cells from the mouse MLN using magnetic bead sorting and examined the mRNA expression of *IL-17*, *IFN-γ*, *IL-4*, and *TGF-β*. The data illustrated that the expression of *IL-17* was significantly increased after treatment with WT EVs (Figure 4(j)). Our results identified a specific DC subset responsive to PMA1-containing EVs, namely cDC2, which migrated from the LP to MLN and activated the TH17 immune response to intensify colitis in mice.

### 2.5. PMA1-containing EVs induce DC maturation and migration and Th17 cell differentiation in vitro

**Figure 5.**
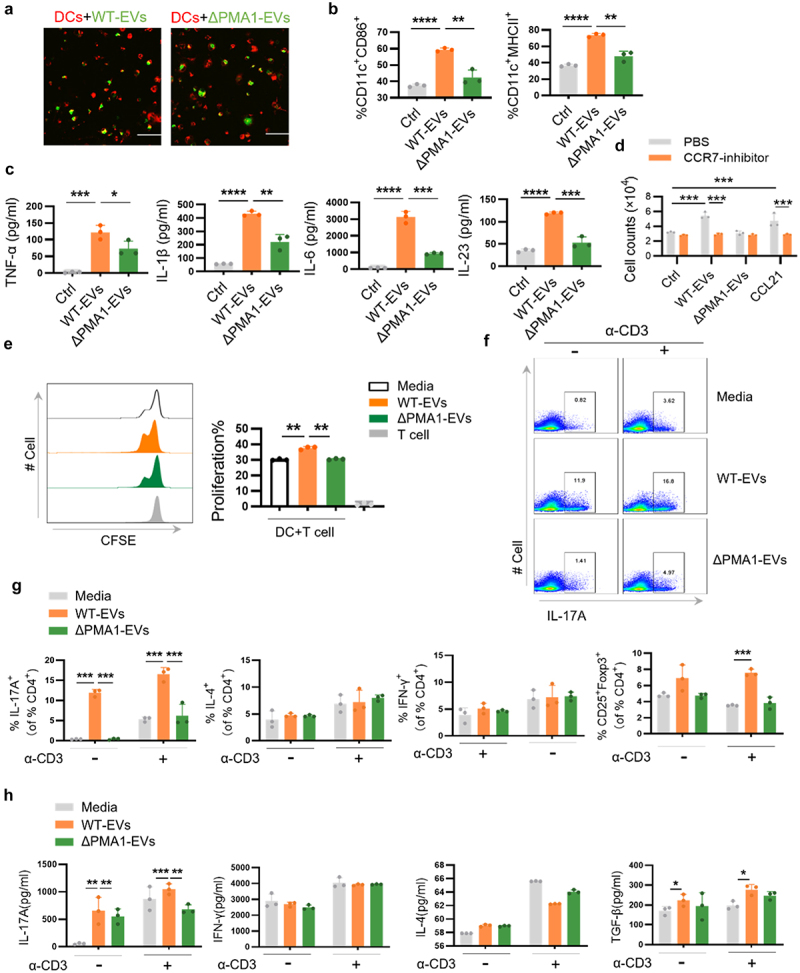
Treatment of DCs with PMA1-containing EVs induces cytokine production, DCs migration, and TH17 cell differentiation.

We further confirmed the immunomodulatory properties of PMA1-containing EVs *in vitro*. Images from confocal microscopy revealed that both WT-EVs and ΔPMA1-EVs (labeled with FITC) could be taken up by BMDCs and appeared as punctuate foci throughout the cytoplasm of cells (Figure 5(a)). EVs were rapidly internalized into DCs in an actin-dependent manner, as treatment of cells with cytochalasin D significantly inhibited the uptake of EVs (Figure S5A – B). The activation markers of DCs (MHCII, CD86) were increased following PMA1-containing EVs stimulation (Figure 5(b)). The protein levels of TNF-α, IL-1β, IL-6, and IL-23 were also significantly upregulated after WT-EVs stimulation (Figure 5(c)), while ΔPMA1-EVs had no significant effect on the activation and cytokine production of DCs (Figure 5(b–c)). We also detected DC migration *in vitro* using Transwell assay. As shown in Figure 5(d), the number of cells that migrated to the underside of the chamber increased after WT-EVs stimulation, while ΔPMA1-EVs were unable to promote DC migration. In addition, the number of migratory DCs was significantly reduced after cosalane (a CCR7 inhibitor) treatment (Figure 5(d)). The BMDCs were then treated with different EVs and cultured with naïve CD4^+^ T cells. The proliferation of CD4^+^ T cells was detected using CFSE staining. WT-EVs-treated DCs induced the proliferation of CD4^+^ T cells, while ΔPMA1-EVs-treated DCs had no significant effect on the proliferation of T cells (Figure 5(e)). We then detected T-cell differentiation using flow cytometry. The frequency of IL-17A^+^ CD4^+^ T cells was markedly increased after co-culture with WT-EVs-treated DCs, while ΔPMA1-EV-treated DCs had no significant effect on the differentiation of TH17 cells (Figure 5(f–g)). We also examined the production of cytokines from T cells. DCs treated with WT-EVs induced more IL-17A production than DCs treated with ΔPMA1-EVs (Figure 5(h)). Collectively, from our *in vivo* and *in vitro* experiments, we found that PMA1-containing EVs induced DC maturation, secretion of cytokines, migration, and specifically induced functional TH17 differentiation.

(a – d) BMDCs were treated with WT-EVs or ΔPMA1-EVs for 24 h. (a) WT-EVs and ΔPMA1-EVs were labeled with FITC (green) and incubated with BMDCs. Cells were fixed and the cell membrane was stained with DiI (red). EVs uptake by BMDCs was observed by confocal microscopy. Scale bar represents 20 μm. (b) The expression of CD86 and MHCII was detected by flow cytometry. (c) The production of TNF-α, IL-1β, IL-6, and IL-23 in the cell supernatant was assessed by ELISA. (d) BMDCs were pretreated with 10 μg/ml cosalane (CCR7 inhibitor) for 1 h, and then cultured in an 8-μm Transwell chamber. The number of cells that migrated below the chamber was detected by flow cytometry. (e – h) BMDCs were stimulated with WT-EVs or ΔPMA1-EVs, and then co-cultured with CD4^+^T cell for 3 days. (e) Proliferation of CD4^+^T cell was measured by flow cytometry. (f – g) The proportion of IL-17A^+^, IFN-γ^+^, IL-4^+^, or Foxp3^+^ CD4^+^ T cells was detected by flow cytometry. (h) The production of IL-17A, IFN-γ, IL-4, and TGF-β in the cell supernatant was assessed by ELISA. Data with error bars represent the mean ± SD. Each panel is representative of at least three independent biological replicates. Scale bars: 50 mm. *P < 0.05, **P < 0.01, ***P < 0.001, ****P < 0.0001 as determined by unpaired Student’s t test.

### 2.6. CARD9 on DCs is required to detect EVs-associated PMA1

**Figure 6.**
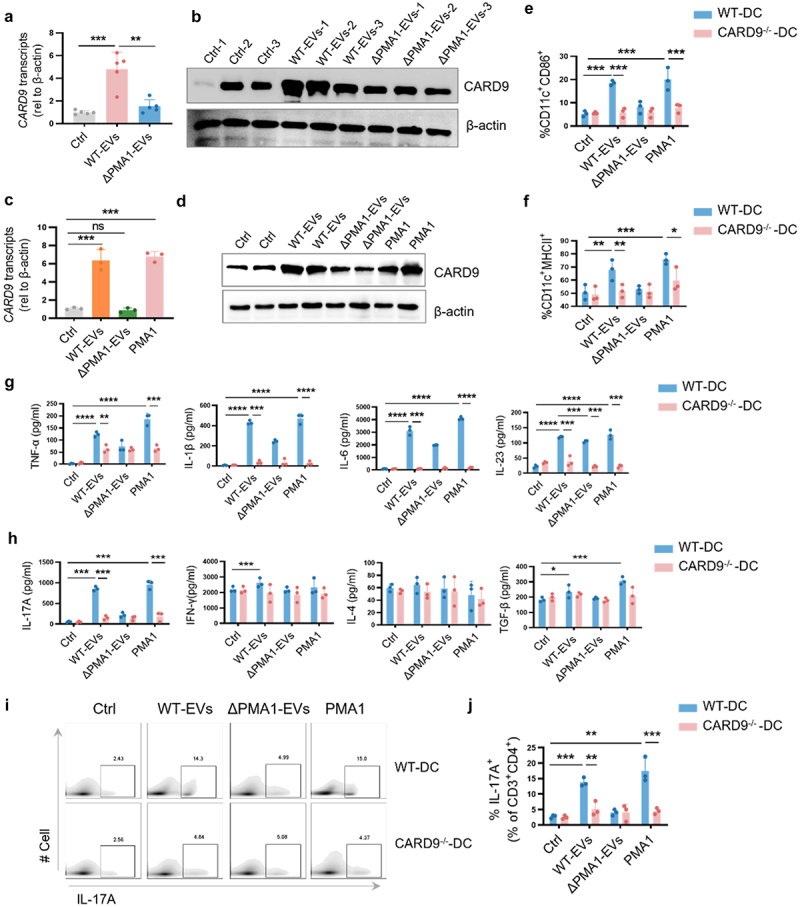
CARD9 on DCs is required to sense EVs-associated PMA1.

CARD9 is an adaptor molecule that recognizes *C. albicans*.^36,37^ However, the role of CARD9 expression in EVs recognition remains unknown. First, we detected the expression of CARD9 in primary cDC2 isolated from mice with colitis treated with WT-EVs and ΔPMA1-EVs. CARD9 mRNA and protein expression was increased after treatment with WT-EVs compared to that in the DSS group, while ΔPMA1-EVs treatment could not up-regulate CARD9 expression (Figure 6(a–b)). Then, we examined the expression of CARD9 in BMDCs after EV and PMA1 stimulation. The results showed that CARD9 gene and protein expression was significantly upregulated after WT-EV or PMA1 protein stimulation (Figure 6(c–d)), while ΔPMA1-EVs could not induce CARD9 expression, suggesting a potential link between PMA1 and CARD9.

(a – b) Mice were treated as described in Figure 3c. The cDC2 subset was separated from the LP by flow sorting. Each dot represents an individual mouse. (a) mRNA expression of *CARD9* was detected by qRCR. (b) The protein level of CARD9 was detected by western blotting. (c – d) BMDCs were stimulated with WT-EVs, ΔPMA1-EVs, or PMA1 protein for 24 h. (c) The mRNA expression of *CARD9*was detected by qPCR, and (d) the protein level of CARD9 was detected by western blotting. (E – g) BMDCs were isolated from WT mice or *CARD9*^*-/-*^ mice, and stimulated with WT-EVs or ΔPMA1-EVs for 24 h. (e) The production of CD86 of DCs was detected by flow cytometry. (f) The production of MHCII of DCs was detected by flow cytometry. (g) The production of TNF-α, IL-1β, IL-6, and IL-23 was assessed by ELISA. (H – J) BMDCs were isolated from WT mice or *CARD9*^*-/-*^ mice, and stimulated with WT-EVs or ΔPMA1-EVs, and then co-cultured with CD4^+^ T cell for 3 days. (h) The production of IL-17A, IFN-γ, IL-4, and TGF-β in the cell supernatant was assessed by ELISA. (i – j) The proportion of IL-17A^+^ T cells was detected by flow cytometry. Data with error bars represent the mean ± SD. Each panel is representative of at least three independent biological replicates. Scale bars: 50 mm. *P < 0.05, **P < 0.01, ***P < 0.001, ****P < 0.0001 as determined by unpaired Student’s t test.

We then studied the role of CARD9 during the pathogenesis of PMA1-containing EVs. BMDCs acquired from wild-type mice (WT-DC) and CARD9-deficient mice (CARD9^−/−^-DC) were pulsed with EVs or purified PMA1 protein. Compared to WT-DC, CARD9^−/−^-DC had no defect in EVs internalization based on both confocal microscopy and flow cytometry (Figure S6A – B). However, compared to WT-DCs, the expression of CD86 and MHCII was impaired in CARD9^−/−^-DC after WT-EVs treatment, ΔPMA1-EVs did not induce maturation of WT-DC and CARD9^−/−^-DC (Figure 6(e–f)). Similar results were found for the secretion of TNF-α, IL-1β, IL-6, and IL-23 in WT-DCs and CARD9^−/−^-DCs (Figure 6(g)). We then treated WT-DCs and CARD9^−/−^-DCs with different EVs and co-cultured them with CD4^+^ T cells. WT-DCs treated with WT-EVs or PMA1 promoted TH17 differentiation and IL-17A production in T cells, while CARD9^−/−^-DC did not support TH17 differentiation and IL-17A production in response to either WT-EVs or PMA1 (Figure 6(h–j) and S6C). Our findings indicate that CARD9-deficient DCs fail to respond to PMA1-containing EVs, demonstrating that CARD9 is a central signaling component in DCs required for commensal fungal-driven TH17 induction and function.

### 2.7. Activation of glycolysis is CARD9-dependent in DCs upon EVs stimulation

**Figure 7.**
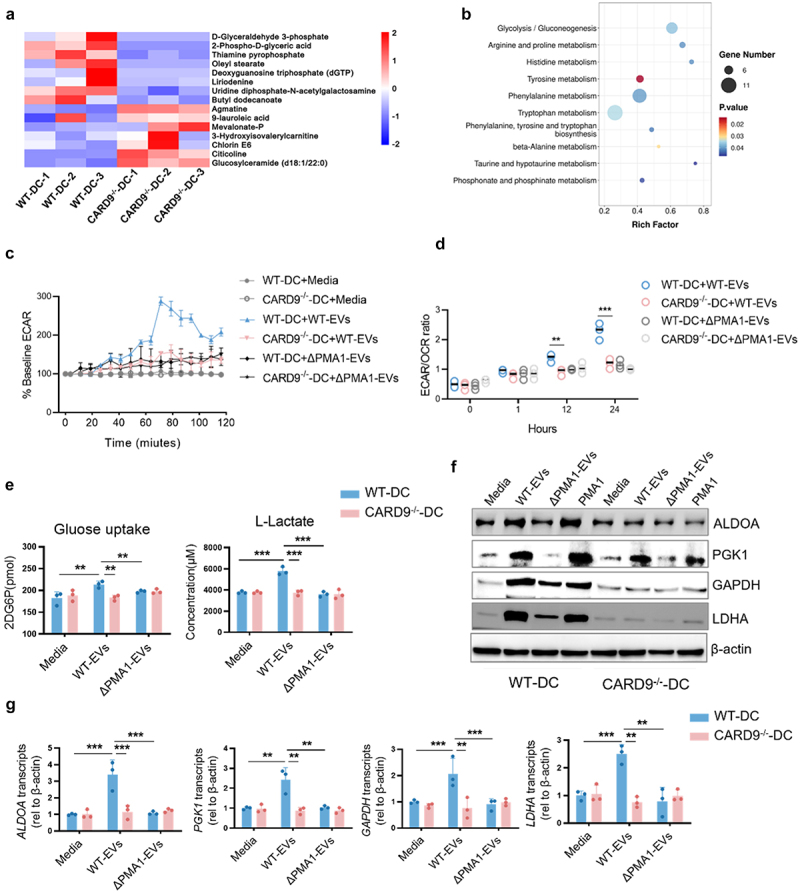
Activation of glycolysis is CARD9 dependent in DCs following EV stimulation.

Given the implication of metabolism in maintaining the immune response of DCs,^38,39^ non-targeted LC/MS metabolomics was used to detect metabolism pathways in WT-DC and CARD9^−/−^-DC after EVs stimulation. PMA1-containing EVs increased glucose metabolites (D-Glyceraldehyde 3-phosphate, 2-phospho-D-glyceric acid, and Thiamine pyrophosphate) and enriched glycolysis pathways in WT-DCs compared to CARD9^−/−^-DCs (Figure 7(a–b)). Real-time changes in ECAR and the ECAR/OCR ratio were also analyzed. As shown in Figure 7(c), WT-EVs strongly promoted the process of glycolysis in WT-DCs but not in CARD9^−/−^-DCs. Meanwhile, ΔPMA1-EVs could not induce glycolysis (Figure 7(c)). Similar results were observed for the ECAR/OCR ratio (Figure 7(d)), glucose uptake, and lactate production in these cells (Figure 7(e)). Furthermore, glycolysis-related proteins were detected using western blotting. As shown in Figure 7(f), PMA1-containing EVs induced aldolase A (ALDOA), phosphoglycerate kinase1 (PGK1), Glyceraldehyde-3-phosphate dehydrogenase (GAPDH), and L-lactate dehydrogenase (LDHA) protein expression in WT-DCs. This induction was impaired in CARD9^−/−^-DCs, suggesting that CARD9 regulates the metabolism of DCs. Similar results were observed for the mRNA expression of glycolytic genes (Figure 7(g)). These results collectively demonstrate that PMA1-containing EVs induce DC glycolysis through CARD9.

(a – b) BMDCs were isolated from WT mice or *CARD9*^*-/-*^ mice stimulated with WT-EVs for 24 h, before performing metabolomic analysis. (a) Heatmaps showing the changed metabolites of the glucose metabolism. (b) Bubble map showing the altered metabolic pathways. (c – g) BMDCs isolated from WT mice or *CARD9*^*-/-*^ mice stimulated with WT-EVs, ΔPMA1-EVs, or PMA1. (c) ECAR of BMDCs for the indicated time. (d) ECAR/OCR in BMDCs for the indicated time. (e) The uptake of glucose was detected by a glucose assay kit and the production of lactate was detected by lactate assay kit. (f) The protein expression of ALDOA, PGK1, GAPDH, and LDHA was detected by western blotting. (g) The mRNA expression of *ALDOA*, *PGK1*, *GAPDH*, and *LDHA* was detected by qPCR. Data with error bars represent the mean ± SD. Each panel is representative of at least three independent biological replicates. Scale bars: 50 mm. *P < 0.05, **P < 0.01, ***P < 0.001 as determined by unpaired Student’s t test.

### 2.8. CARD9 mediates glycolysis by interacting with GAPDH

**Figure 8.**
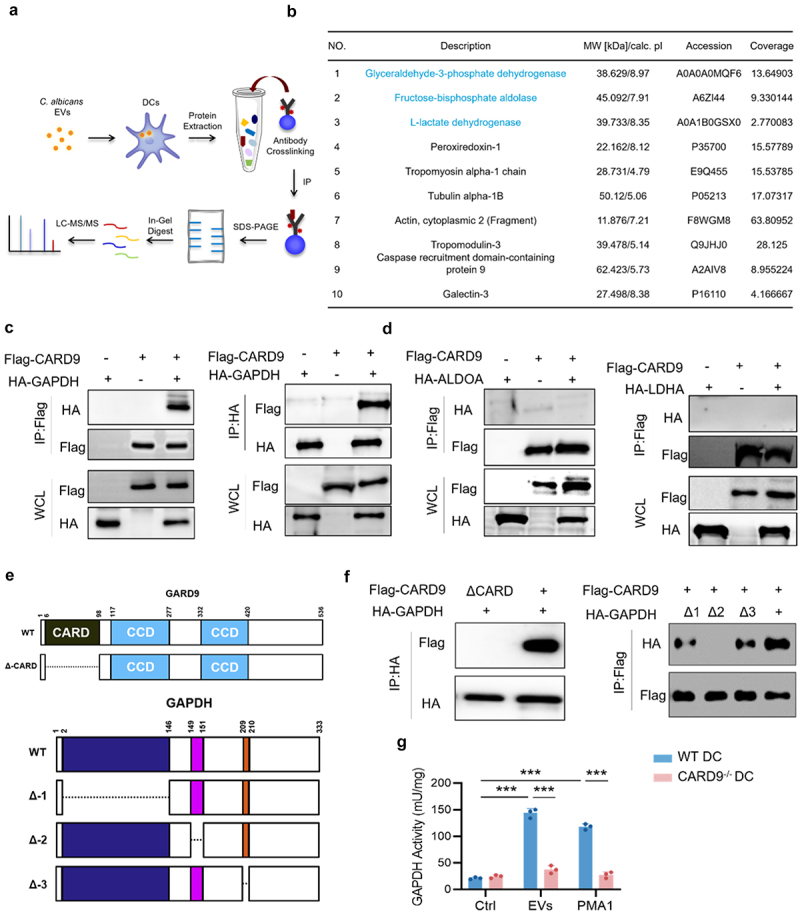
CARD9 mediates glycolysis via GAPDH.

To investigate whether CARD9 recruits glycolytic proteins during PMA1-containing EVs in DCs, a co-immunoprecipitation (CoIP) – MS strategy was established to analyze the interacting proteins of CARD9 (Figure 8(a)). After excluding all background interfering proteins, three proteins of the glycolysis pathway (GAPDH, ALDOA, and LDHA) were identified as potential CARD9 interacting proteins (Figure 8(b)). The interaction between CARD9 and GAPDH was further confirmed using the ectopic expression of Flag-CARD9 with HA-GAPDH, HA-ALDOA, or HA-LDHA in HEK293T cells (Figure 8(c–d)). Because CARD9 contains N-terminal CARD domains 1 (aa 6–98), CCD domain 2 (aa 117–277), and CCD domain 3 (aa 332–420), and GAPDH contains three domains (domain 1, aa 2–146; domain 2, aa 149–151; domain 3, aa 209–210), protein mutants with a single missing domain were constructed (Figure 8(e)). Results of the CoIP experiment showed that CARD9 interacted with endogenous GAPDH through its CARD region, whereas GAPDH interacted with CARD9 through its domain 2 (aa 2–146) (Figure 8(f)). To confirm the endogenous interaction between CARD9 and the enzyme activity of GAPDH, WT-DC and CARD9^−/−^-DC were treated with WT-EVs or PMA1 protein. As shown in Figure 8(g), GAPDH activity was significantly decreased in CARD9^−/−^-DC upon WT-EVs treatment compared to that in WT-DCs. These data suggest that the CARD domain of CARD9 could recruit GAPDH upon PMA1 stimulation, whose enzyme activity was activated and the process of glycolysis was therefore activated.

(a – b) BMDCs isolated from WT mice or *CARD9*^*-/-*^ mice stimulated with WT-EVs for 24 h. (a) CARD9-binding proteins were analyzed by the CoIP-MS strategy. (b) LC-MS/MS analysis of CARD9-binding proteins, part of the interacting proteins is shown. (c) HEK293T cells transfected with Flag-tagged CARD9 and HA-tagged GAPDH. After 48 h, cell lysate was used for IP with anti-Flag (left) or anti-HA (right) and IB with anti-Flag or anti-HA. (d) HEK293T cells transfected with Flag-tagged CARD9 and HA-tagged ALDOA (left) or Flag-tagged CARD9 and HA-tagged LDHA (right). Cell lysate was used for IP with anti-Flag and IB with anti-Flag or anti-HA. (e) Protein domain schematic diagram of CARD9 and its domain mutants (up), and protein domain schematic diagram of GAPDH and its domain mutants (down). (f) HEK293T cells transfected with Flag-CARD9 WT or its mutant and HA-GAPDH, followed by IP with HA (left). HEK293T cells transfected with Flag-CARD9 and HA- GAPDH WT or its mutant, followed by IP with Flag (right). (g) WT or CARD9^−/−^ BMDCs after stimulation with WT-EVs or PMA1 protein for 24 h. GAPDH enzymatic activity in were detected by the Glyceraldehyde 3 Phosphate Dehydrogenase Activity Assay Kit. Data with error bars represent the mean ± SD. Each panel is a representative experiment of at least three independent biological replicates. Scale bars: 50 mm. *P < 0.05, **P < 0.01, ***P < 0.001 as determined by unpaired Student’s t test.

**Figure 9.**
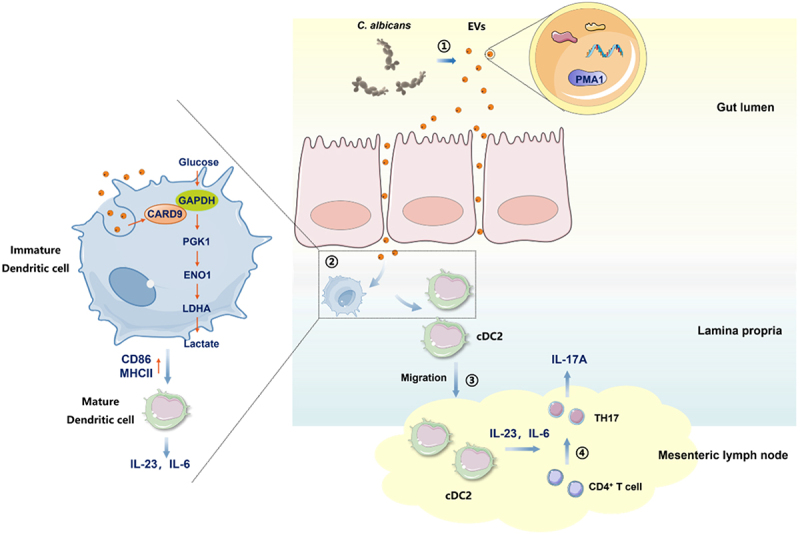
Graphic illustration of the mechanism by which *C. albicans* EVs release PMA1 to mediate DC immune responses and colitis progression.

In this study, we demonstrated that PMA1 is a potential virulence factor responsible for the pathogenesis of *C. albicans*-associated colitis. PMA1 was found to be packaged into EVs from *C. albicans* and internalized into DCs. PMA1-containing EVs promoted the maturation, migration, and cytokine production of DCs. Specifically, the cDC2 subgroup in the intestinal LP migrated to the MLN under EV stimulation and induced TH17 differentiation, promoting the development of colitis. Mechanically, PMA1 induced CARD9 expression in DCs, which could recruit and activate the enzyme activity of GAPDH, thereby promoting glycolysis in DCs (Figure 9).

## 3. Discussion

Recently, Li et al. discovered a rich genetic diversity of opportunistic *C. albicans* strains in the colonic mucosa of patients with IBD.^7^ This finding suggests that *C. albicans* may be characteristic in patients with IBD, rather than merely increasing numbers. We conducted gene expression sequencing analysis of intestinal C. albicans in healthy individuals and patients with colitis. The results showed that PMA1 expression was significantly upregulated in patients with colitis. After knocking out PMA1 in C. albicans, we found that the pathogenic effect of C. albicans was eliminated. In contrast to previous understanding of the inflammatory factors of *C. albicans*, such as glucan and zymosan, we identified a new pathogenic factor of *C. albicans* in colitis. We identified PMA1 as a symbiotic factor of *C. albicans*, which deteriorates inflammatory disease in animals by inducing an immune response.

However, no previous study has elucidated the mechanism by which PMA1 is delivered to the immune system. The studies described here illustrate that EVs released from the fungal surface are sufficient to mediate inter-kingdom interactions between *C. albicans* and the immune system by delivering PMA1 to DCs. Oral treatment of mice with EVs can aggravate experimental colitis, whereas EVs from ΔPMA1-*C. albicans* are unable to cause more severe histopathology and proinflammatory cytokine production. Our work reveals a paradigm in which an opportunistic pathogen of the human microbiota selectively delivers pathogenic molecules to its host rather than simply through direct contact.

In response to sampling both self and foreign antigens, DCs interact with various immune cells to coordinate diverse biological responses. The presentation of microbial molecules to CD4^+^ T cells is a key component of an effective immune response to infection. We previously found that PMA1 induces the immune response of DCs and TH17 cells (data not shown). Here we show that EVs internalized by DCs induce cytokine production and migration and drive the development of IL-17A-producing TH17 cells, whereas ΔPMA1-EVs do not promote TH17. Furthermore, the response of EVs is DC-dependent, as treatment of T cells alone with EVs (containing PMA1) cannot promote IL-17 production by CD4^+^ T cells. The subgroups of DCs in the gut have been clearly delineated, and several studies have unraveled different functionalities of DC subsets within the intestine, especially in inflammatory environments.^40^ Indeed, the commensal strain Cryptosporidium tyzzeri (Ct-STL) elicits a cDC1-dependent TH1 response that promotes intestinal homeostasis. In addition, colitis remission of Rag1^−/−^ irf4^−/−^ T cell receptors may be due to regulation of cDC2, which affects TH17 differentiation.^41^ It will be interesting to elucidate the subset of DCs that respond to PMA1/EVs. Our results showed that cDC2 in the LP are specifically activated by PMA1-containing EVs and can migrate to MLNs to contribute to inducing TH17 differentiation.

The biological context by which DCs contact PMA1 during colonization by *C. albicans* remains unclear, and further studies involving *in vivo* metabolic labeling of PMA1 are required to distinguish when, where, and how each cell type ‘sees’ PMA1. However, we speculate that DCs internalize PMA1 associated with EVs, which traffic through the endocytic pathway to contact CD4^+^ T cells. This notion is supported by evidence showing that PMA1 is presented to CD4^+^ T cells by MHCII after internalization and processing in the endosome. What is clear from our results is that PMA1, in the context of EVs, directly activates DCs and not T cells (Figure 2(i)). This interaction leads to the immune activation of DCs, which enhances TH17 function and promotes the development of inflammatory disease.

Immune cells recognize microbial ligands through pattern recognition receptors to initiate immunologic responses. Previous studies have identified CARD9 as being necessary for Dectin-1-mediated *C. albicans* recognition,^42^ as well as for IL-17 production in T helper cells.^43^ Our study shows that DC expression of CARD9 is necessary for the induction of inflammatory responses by EVs. This phenotype is largely dependent on PMA1, although EVs contain other molecules (perhaps lipoproteins) that also increase TNF-α expression through CARD9. Fluorescent labeling of EVs showed that they could be endocytosed by DCs, although CARD9 does not participate in this process. Our data showed that the production of TNF-α, IL-1β, IL-6, and IL-23 was reduced in CARD9^−/−^ DCs, and the absence of CARD9 affected the migration ability of DCs. In addition, the absence of CARD9 in EVs-treated DCs leads to a defect in the induction of IL-17A production by CD4^+^ T cells. CARD9, a component of the CBM complex, has been shown to activate the NF-κB pathway during fungal recognition.^44^ In this study, we demonstrated a new function of CARD9 in the metabolic process. We found that CARD9 knockout reduced the glycolytic metabolic function of DCs. Additionally, the expression levels of genes and proteins of key enzymes in glycolysis pathways, such as GAPDH, were significantly down-regulated in CARD9^−/−^ DCs. As an adaptor protein, CARD9 interacts with other proteins, such as BCL10 and rubicon, via the caspase-recruitment domain (CARD).^45,46^ Further study revealed that GAPDH in the glycolysis pathway directly interacts with CARD9. Our study illustrates a novel mechanism by which CARD9 recognizes fungal molecules in DCs. After the *C. albicans* virulence molecule PMA1 is carried into cells through EVs, it activates the glycolytic metabolism of DCs through CARD9, thereby affecting the secretion and migration of DCs.

Our study revealed that *C. albicans* actively delivers PMA1 through EVs, revealing a previously unrecognized mechanism by which *C. albicans* can deliver its virulence molecules to the host, contributing to its infection and disease development. These results show that fungal microbiota molecules can mediate the critical balance between health and disease, harnessing the immunomodulatory capacity of pathogenic factors such as PMA1. Interference with these virulent molecules may provide therapeutic agents for human inflammatory disorders based on entirely novel biological principles.

## 4. Star methods

### 4.1. Patients

Fecal samples of healthy people (*n* = 5) and patients diagnosed with UC (*n* = 5) were obtained from Nanjing University Affiliated Drum Tower Hospital between July 2019 and May 2020. The demographic results of the controls and patients are presented in Table EV1. All research involving human participants was approved by the ethics committee of the ‘‘Medical School of Nanjing University.’’ Written informed consent was obtained from all subjects.

### 4.2. Isolation and culture of *C.* *albicans* strains

To isolate *C. albicans* from human samples, 50–300 mg of feces was resuspended into 500 μl phosphate buffered saline (PBS) and serial dilutions were made, followed by plating 100 μl onto a YPD agar plate with antibiotics (1% yeast extract, 2% peptone, 2% D-glucose, 1.5% agar, 100 μg/ml gentamicin, and 100 μg/ml chloramphenicol). The plates were incubated at 30°C for 48 h. At least 10 colonies were collected from each plate. Each colony was assessed by qPCR using specific primers for the *C. albicans* ITS gene. Next, RNA sequencing was performed to analyze gene expression in a random colony of *C. albicans* (GEO record: GSE230146). To evaluate the mRNA expression of *PMA1* in *C. albicans* isolates, five *C. albicans* colonies from each sample were verified using qPCR.

The *C. albicans* strain (SC5314) was obtained from Dr. Xin Lin (Tsinghua University, Beijing, China). All *C. albicans* strains were grown in YPD broth with antibiotics (1% yeast extract, 2% peptone, 2% D-glucose, 100 μg/ml gentamicin and 100 μg/ml chloramphenicol) and incubated at 30°C with shaking at 150 rpm for 20 h. The cell density was determined by measuring the optical density at 600 nm using a microplate reader (Molecular Devices). Cells from YPD broth were collected via centrifugation, washed twice with PBS, and adjusted to the required cell density.

### 4.3. Construction of ΔPMA1-*C.* *albicans* strains

Deletion of PMA1 in *C. albicans* was performed using CRISPR-Cas9 genome editing technology, as previously described.^47^ In brief, *Candida*-optimized Cas9-based plasmids for PMA1 deletion were constructed and transfected into *C. albicans* haploids (obtained from Professor *Rebecca S. Shapiro*, University of Guelph, Guelph, ON, Canada). An optimized mating strategy was used to generate homozygous single- and double-gene diploid mutants. The deletion of PMA1 in *C. albicans* was confirmed using gel electrophoresis (Figure S2A).

### 4.4. EVs purification and labeling

Isolation of EVs from *C. albicans* was performed as previously described.^48^ In brief, *C. albicans* and ΔPMA1-*C. albicans* culture medium were subjected to sequential centrifugation steps (4000 ×g, 15 min and 15,000 ×g, 30 min, at 4°C). Cell-free supernatants were then concentrated approximately 20-fold using an Amicon ultrafiltration system (cutoff, 100 kDa, Millipore) and then centrifuged at 100,000 ×g for 1 h at 4°C. The supernatants were then discarded, and the EV-containing pellets were washed twice with 0.1 M PBS at 100,000 ×g for 1 h at 4°C. The EVs were then characterized by SEM, TEM, and nanoflow. The total protein recovered from each EV preparation was normalized to the OD600 of the culture at harvest.

FITC-labeled EVs were prepared as previously described.^49^ EVs were fluorescently labeled by diluting 1:1 with FITC (1 mg/ml in 50 mm Na_2_CO_3_ and 100 mm NaCl, pH 9.2) and incubating for 1 h at 25°C. EVs were pelleted (52,000 ×g, 30 min) and washed three times with sterile 50 mm HEPES at pH 6.8. FITC-labeled EVs were resuspended in Dulbecco’s PBS supplemented with 0.2 M NaCl and were checked for sterility and protein concentration.

### 4.5. Mice

C57BL/6 mice were purchased from Nanjing Cavans Biotechnology Co., Ltd. *CARD9*^*-/-*^ mice were generated and bred in our laboratory as previously described.^50^ Mice were raised in sterile facilities at the Medical College of Nanjing University. All animal experiments were performed according to NIH guidelines for “The Care and Use of Laboratory Animals” and were approved by the Institutional Committee of Animal Care and Use at the Medical College of Nanjing University. The authorization number from the committee is SYXK2019–0056.

For the colitis model, mice (6–8 weeks old) were fed 2.5% DSS (MW36–50 kDa, MP Biomedicals, USA) dissolved in drinking water for 7 consecutive days. For *C. albicans*-treated experiments, mice were pretreated with an antibiotic cocktail (Abx) of 1 mg/mL each of ampicillin (sodium salt, SIGMA), neomycin sulfate (Fisher), metronidazole (Fisher), 0.5 mg/mL vancomycin hydrochloride (Fisher), and 500 mg/kg fluconazole (MCE) for 7 days, and then orally treated with *WT-C.a*, *ΔPMA1-C.a*, *C. albicans* isolates from healthy people (*H-C.a*), or from *C. albicans* isolates from patients with UC (*UC-C.a*) every other day (1 × 10^8^ CFU) during DSS processing. For the EV-treated experiments, mice were treated with WT-EVs or ΔPMA1-EVs (5 μg) every other day during DSS processing. Before gavage, mice were starved for 5 h to reduce gastric acid production. On day 8, mice were sacrificed and colon tissues were collected.

For the *C. albicans* treated alone experiments, mice were pretreated with an antibiotic cocktail (Abx) for 7 days and then gavaged every other day with PBS, *H-C.a*, *UC-C.a*, *WT-C.a*, or *ΔPMA1-C.a* (1 × 10^8^ CFU) without DSS processing for another 7 days. For EVs treated alone experiments, mice were gavaged every other day with PBS, WT-EVs, or ΔPMA1-EVs (5 μg) without DSS processing for 7 days. The body weight of each mouse was measured daily.

We examined male and female animals, and similar findings were reported for both sexes.

### 4.6. Disease activity index

Individual scores were combined to generate the DAI, which was calculated daily for each mouse. The maximum score was 12 based on a 0–4 scoring system for the following parameters: 0, no weight loss, normal stool consistency, and no blood in stool; 1, 1–5% weight loss, loose stools, and blood in stools; 2, 5–10% weight loss, watery diarrhea, and blood in stools; 3, 10–20% weight loss, slimy diarrhea, with a little blood; and 4, > 20% weight loss, severe watery diarrhea with blood, and gross bleeding.

### 4.7. Evaluation of colonic tissue

The colon length was measured in each mouse. Colon tissue was harvested, fixed in neutral 10% buffered formalin, embedded in paraffin, sectioned, and stained with hematoxylin and eosin (H&E). All histology was evaluated in a blinded manner by two pathologists using a comprehensive scoring system. H&E-stained slides were scanned and digitally scored using a slide imaging system (Hamamatsu NanoZoomer Slide Scanner 2.0HT). The colitis score was generated based on a modification of an approach described previously.^51^ Briefly, the number and distribution of mucosal polymorphonuclear cells, mononuclear cells, and epithelial cell damage were scored from 0 to 4. The submucosa was scored for cellular infiltrates from 0 to 3 while the thickening and cellular infiltrates in the muscularis were scored from 0 to 2.

### 4.8. Isolation of colonic tissues

The colonic LP lymphocytes were isolated as previously described.^52^ Briefly, the colons were cut open longitudinally and flushed with ice-cold PBS. The colons were cut into 1-cm pieces and incubated for 20 min in 10 mm dithiothreitol (DTT, Sigma) with gentle shaking, followed by two incubations for 20 min in 20 mm EDTA (Sigma). The supernatants were removed, and the remaining tissue was incubated in 1 mg/ml Collagenase D (Sigma), 0.25 U/ml Dispase (Roche), and 0.5 mg/ml DNase I (Worthington). Cells were filtered through a 70-μm cell strainer (BD Falcon) and separated using a 40/80% (v/v) Percoll (GE Healthcare) density gradient. Cells were washed before flow cytometric staining.

MLNs were acquired and processed by grinding tissues through a 70-μm cell strainer (BD Falcon) to generate single-cell suspensions.

### 4.9. Flow cytometry and intracellular cytokine staining

To detect the proportion of immune cells, single-cell suspensions were first stained for live/dead using a Fixable Viability Kit (Zombie Aqua, Biolegend) and then stained with the following antibodies (Biolegend) for 15 min at room temperature: CD4 (PE, #100407), CD8 (PE, #100707), CD11c (FITC, #506907), CD11b (FITC, #101205), F4–80 (PE, #123109), CD3 (FITC, #107607), CD86 (BV510, #105039), MHCII (PE, #107607), CD25 (APC, #162105), CD45 (AF700, #103128), CD14 (FITC, #123307), CD16 (FITC, #158008), CD64 (FITC, #139316), XCR1 (BV421, #148216), SIRPα (APC, #144014), B220 (BV510, #103248), CD206 (AF700, #126419), CCR7 (PE, #120105). For nuclear staining, cells were fixed and permeabilized with the Foxp3/Transcription factor buffer kit (eBioscience) and then stained with Foxp3 (BV421, #126419). For intracellular cytokine staining, cells were pretreated with 1 μl 250 × PMA/Ionomycin, mixed, and cultured for 4–6 h in a 5% CO_2_ incubator at 37°C. For intracellular staining, cells were fixed and permeabilized with the Cyto-Fast Fix/Perm Buffer Set (eBioscience). Intracellular staining was performed using IL-17A (FITC, #506907), IFN-γ (PE, #505807), and IL-4 (BV605, #504125).

### 4.10. Generation of BMDCs and co-culture systems

Bone marrow progenitor cells isolated from the femurs of WT or *CARD9*^*-/-*^ mice were differentiated into BMDCs in the presence of 20 ng/ml GM-CSF (Miltenyi) and 10 ng/ml IL-6 in modified RPMI 1640 media (10% fetal bovine serum, 50 U/ml penicillin, 50 μg/ml streptomycin, 2 mm L-glutamine, 1 mm sodium pyruvate, 1 mm HEPES, non-essential amino acids, and β-mercaptoethanol) for 6 days. BMDCs were pulsed with PBS, WT-EVs, ΔPMA1-EVs (10, 50, or 100 μg/ml), and purified PMA1 protein (1, 10, or 100 μg/ml) for 24 h. The purified PMA1 protein of *C. albicans* was obtained via gene synthesis and protein expression, and was completed by Zoonbio Biotechnology Co., Ltd., China. Supernatants were collected for enzyme-linked immunosorbent assay (ELISA) analysis of TNF-α, IL-1β, IL-6, and IL-23 using commercially available kits (eBiosciences). In some experiments, BMDCs were pre-treated with 5 μM Cytochalasin D for 1 h before the addition of FITC-labeled EVs.

For the DC-T cell co-culture experiment, BMDCs were pulsed with PBS, WT-EVs, ΔPMA1-EVs (10, 50, or 100 μg/ml), purified PMA1 (1, 10, or 100 μg/ml), or lipopolysaccharide (10 μg/ml) for 24 h. BMDCs were then washed and co-cultured with splenic CD4^+^ T cells (CD4^+^ T Cell Isolation Kit, Miltenyi Biotec, #130-104-454) at a ratio of 1:10 (DC:CD4^+^ T cells), with or without α-CD3 (2 μg/ml). After 3 days of co-culture, T-cell proliferation was analyzed by flow cytometry. Supernatants were collected to analyze IL-17A, IFN-γ, IL-4, and TGF-β using commercially ELISA kits (eBiosciences). For intracellular cytokine staining, cells were re-stimulated with 10 ng/ml PMA (Sigma) and 1 μg/ml Ionomycin (Calbiochem) in the presence of GolgiPlug (BD Biosciences) for 4 h and stained with specific antibodies and a viability dye.

### 4.11. Cell migration experiments

Subsets of cDC2 were isolated from the LP in each mouse using flow cytometry. 1 × 10^5^ cDC2 were spread in a 24-well plate with an 8-μm Transwell chamber, and 600 μl medium was added to the lower layer. After 24 h, the lower cells were collected and counted. Similarly, 1 × 10^5^ BMDCs were spread in a 24-well plate with an 8-μm Transwell chamber, and 600 μl medium was added to the lower layer. Cells were pretreated with 10 μg/ml cosalane (CCR7 inhibitor) for 1 h and then treated with WT-EVs (100 μg/ml), ΔPMA1-EVs (100 μg/ml), PMA1 (10 μg/ml), or CCL21 (1 nM). After 24 h, the lower cells were collected and counted.

### 4.12. RNA extraction and RT-PCR

RNA was extracted from colonic tissues and BMDCs using an RNeasy Mini Kit according to the manufacturer’s protocol (Qiagen). Next, 1 μg RNA was reverse-transcribed using an iScript cDNA Synthesis Kit (Bio-Rad) and diluted to 10 ng/μl based on the input concentration of total RNA. The primers used in this study are shown in Table EV2. Real-time PCR was performed on cDNA using an ABI PRISM 7900 hT (ThermoFisher), with β-actin used as a housekeeping gene.

### 4.13. Western blotting, immunoprecipitation, and Co-IP

WT-DC and *CARD9*^*-/-*^-DC (1 × 10^6^) were treated with WT-EVs (100 μg/ml), ΔPMA1-EVs (100 μg/ml), or PMA1 (10 μg/ml) for 24 h. Samples were analyzed on 4–20% Tris-Glycine gels (Novex), and proteins were transferred to Immobilon-P PVDF membranes (EMD Millipore). The membrane was blocked in 5% nonfat dry milk and probed with the following antibodies: CARD9 (Cell Signaling Technology, #77568), GAPDH (Cell Signaling Technology, #2118), PGK1 (Cell Signaling Technology, #63536), HA-Tag (Cell Signaling Technology, #3724), FLAG (Cell Signaling Technology, #14793), or β-actin (Cell Signaling Technology, #4970), ENO1(Invitrogen, #PA5–21387), and LDHA (Invitrogen, #PA5–27406) with a horseradish peroxidase (HRP) – conjugated goat α-rabbit secondary antibody. The chemiluminescent signal was detected using the Gel DocTM XR^+^ System (Bio-Rad).

IP and Co-IP experiments were conducted using a Pierce Classic Magnetic IP/Co-IP Kit (Thermo Scientific, #88804).

### 4.14. LC-MS/MS and data analysis

WT-DC and *CARD9*^*-/-*^-DC (1 × 10^6^) were treated with WT-EVs (100 μg/ml) for 24 h. Metabolomic data analysis was performed by Shanghai Luming Biological Technology Co., Ltd. (Shanghai, China). An ACQUITY UPLC I-Class plus (Waters Corporation, Milford, USA) equipped with a Q-Exactive mass spectrometer equipped with a heated electrospray ionization (ESI) source (Thermo Fisher Scientific, Waltham, MA, USA) was used to analyze the metabolic profiling in both ESI-positive and ESI-negative ion modes. An ACQUITY UPLC HSS T3 column (1.8 μm, 2.1 × 100 mm) was used in both positive and negative modes. The binary gradient elution system consisted of (A) water (containing 0.1% formic acid, v/v) and (B) acetonitrile (containing 0.1% formic acid, v/v), and separation was achieved using the following gradient: 0.01 min, 5% B; 2 min, 5% B; 4 min, 30% B; 8 min, 50% B; 10 min, 80% B; 14 min, 100% B; 15 min, 100% B; 15 min, 5% B, and 16 min, 5% B. The flow rate was 0.35 mL/min, and the column temperature was 45°C. All samples were maintained at 4°C during analysis. The mass range was 100–1,000 m/z 100 to 1,000. The resolution was set to 70,000 for full MS and 17,500 for HCD MS/MS scans. The collision energy was set at 10, 20, and 40 eV. The mass spectrometer was operated as follows: spray voltage, 3800 V (+) and 3200 V (−); sheath gas flow rate, 35 arbitrary units; auxiliary gas flow rate, 8 arbitrary units; capillary temperature, 320°C; Aux gas heater temperature, 350°C; and S-lens RF level, 50.

### 4.15. Metabolism assays

WT-DC and *CARD9*^*-/-*^-DC (1 × 10^6^) were treated with WT-EVs (100 μg/ml, glucose-free medium) for 2 h. Next, 5 μl of medium containing 4.5 g/L glucose was added to each well, and cells were incubated at 37°C for another 1 h. The concentrations of lactic acid and glucose in the medium were measured using a lactate assay kit (Sigma) and glucose assay kit (Sigma), respectively. The extracellular acidification rate (ECAR) and oxygen consumption rate (OCR) were measured using a Metabolic Flux Analyzer (Seahorse Bioscience, North Billerica, MA 24XP and/or 96XP).

### 4.16. Proteomic data analysis

For proteomic analysis of WT-EVs, ΔPMA1-EVs, and EVs-free supernatant, the proteins of WT-EVs and ΔPMA1-EVs were extracted by protein cracking solution, and the protein of the EV-free supernatant was concentrated using an Amicon ultrafiltration system (cutoff, 100 kDa, Millipore). After SDS-PAGE, the gel was dyed with Coomassie bright blue. For the proteomic analysis of the CARD9 interacting protein, BMDCs were treated with 100 μg/ml *C. albicans* EVs, and CADR9 IP antibody was used to conduct IP experiments (Pierce Classic Magnetic IP Kit, Thermo Scientific, #88804). The proteomic data analysis was performed using liquid chromatography-mass spectrometry by Shanghai Luming Biological Technology co., Ltd. (Shanghai, China).

### 4.17. GAPDH activity assay

Intracellular GAPDH activity was measured using a GAPDH Activity Assay Kit (Biovision, No. K680–100). WT-DC and CARD9^−/−^-DC (5 × 10^4^) were plated in 24-well plates and treated with WT-EVs (100 μg/ml) and purified PMA1 (100 μg/ml) for 48 h. Cells were washed and rapidly homogenized with 100 μl GAPDH Assay Buffer and kept on ice for 10 min. Then, cells were centrifuged at 10,000 ×g and 4°C for 5 min and 5 μl supernatant per well for the assay was added. The standard was added to a series of wells in a 96-well plate to generate the NADH standard. The final mixture was incubated at 37°C for 60 min. The absorbance of each sample at 450 nm was measured at 0 and 60 min, and the inhibition rates were calculated according to the absorbance values and NADH standard curve. The relative GAPDH activity was normalized according to the respective cell lysate protein concentrations.

### 4.18. Statistical analysis

The results are expressed as the mean ± SD of three independent experiments, and each experiment included triplicate sets. The data were statistically evaluated using one-way ANOVA followed by Dunnett’s test between the control and multiple-dose groups. A *p* value < 0.05 was considered statistically significant.

## Supplementary Material

Supplementary material.docx

## Data Availability

The data are contained in the article and supporting information.
