# Supplementary material for: PMA1-containing extracellular vesicles of Candida albicans triggers immune responses and colitis progression
Source: Gut Microbes. 2025 Jan 31;17(1):2455508. doi: 10.1080/19490976.2025.2455508 (PMC11792855; doi:10.1080/19490976.2025.2455508)
Supplement: Supplementary material.docx [file KGMI_A_2455508_SM7924.docx]

**Supplementary material**


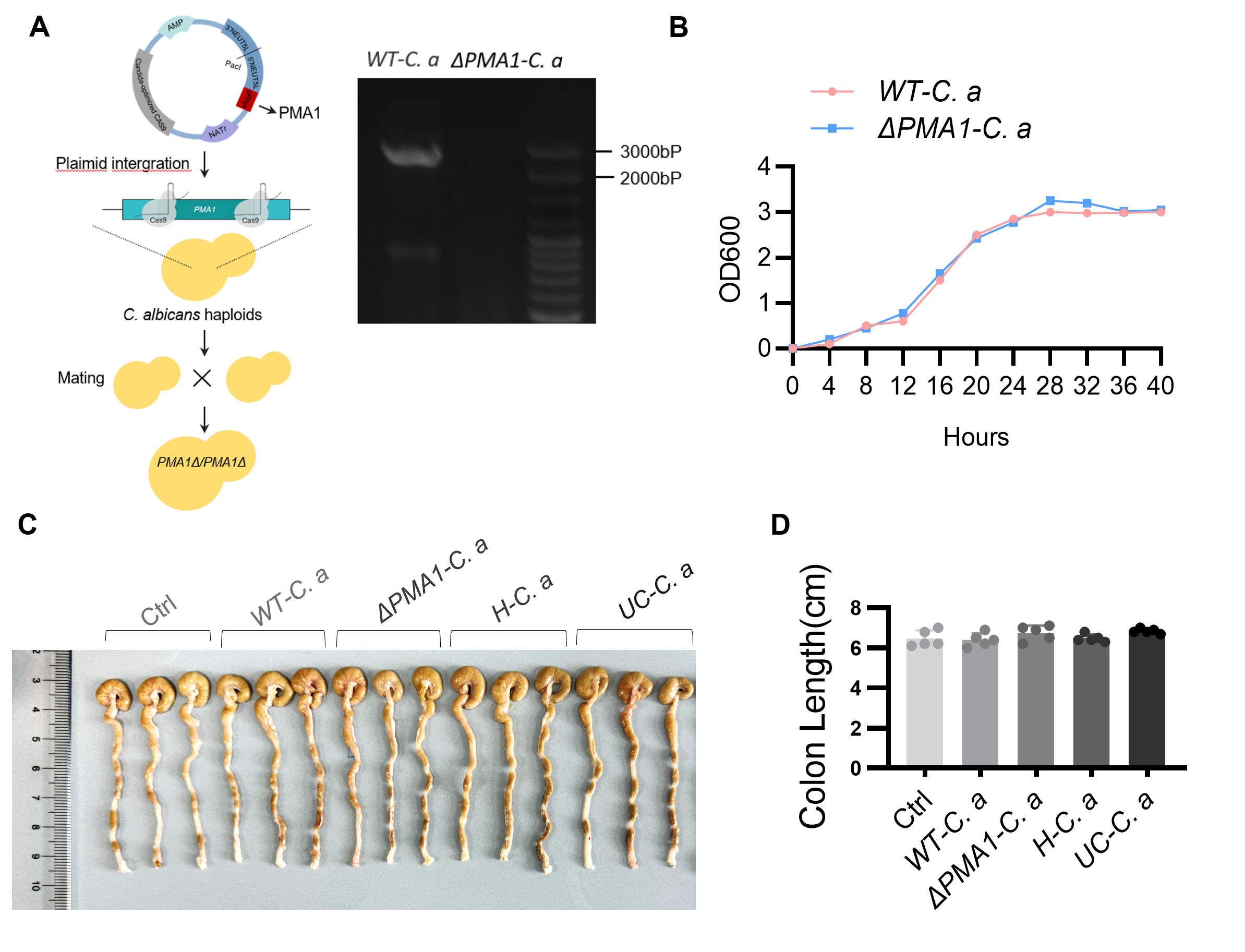


**Figure S1. *C. albicans* PMA1 aggravates colitis.**

(A) Flowchart for building *ΔPMA1-C.a*. (B) OD600 values of *WT-C.a* and *ΔPMA1-C.a* were detected under different culture times. (C-D) Mice were gavaged every other day with PBS, 1×10^8^ WT-*C.a*, *ΔPMA1-C.a*, *H-C.a* or *UC-C.a* (n=5 of each group). Colon length were measured, each dot represents an individual mouse.


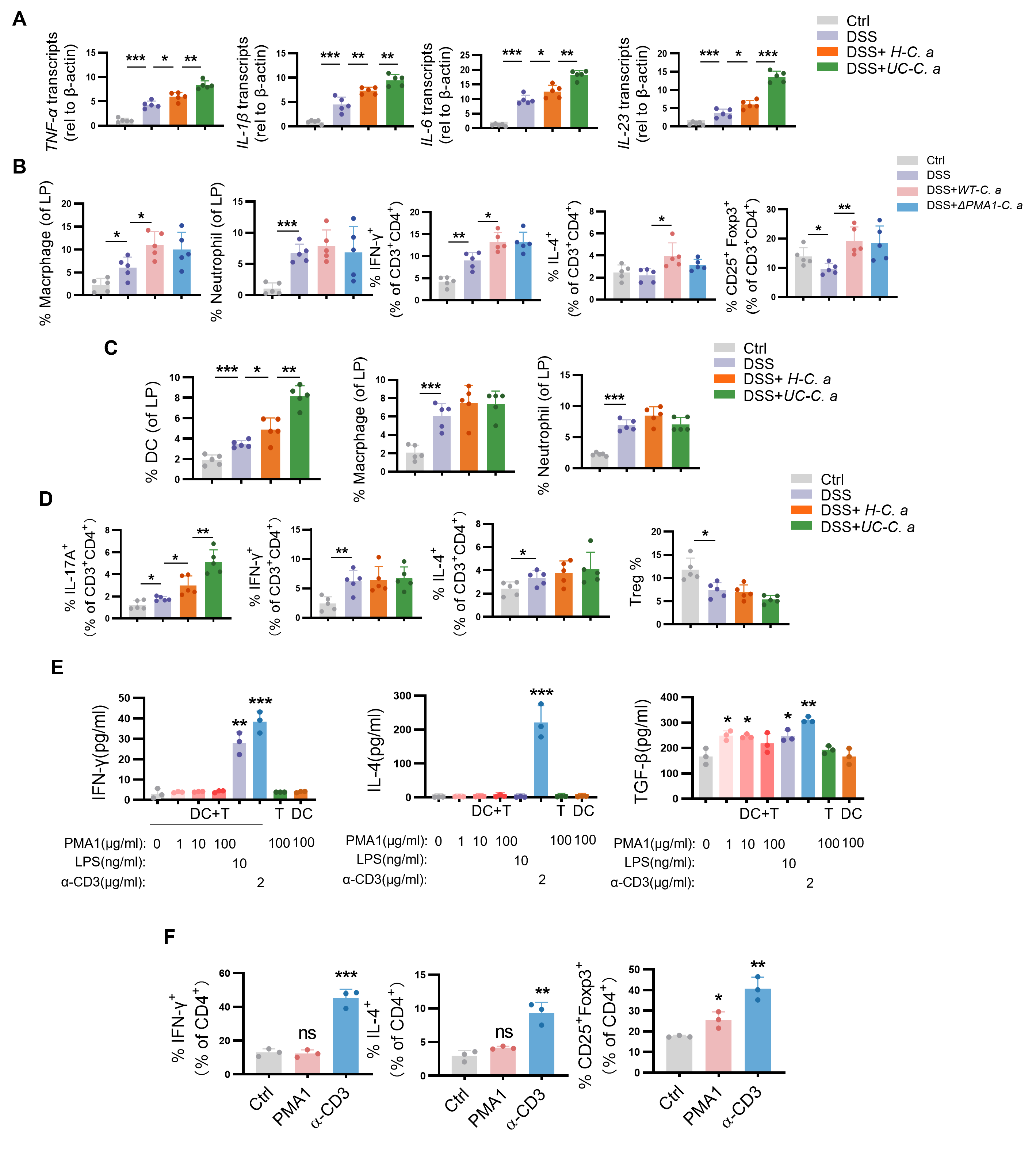


**Figure S2. PMA1 is recognized by DCs, with consequent T cell activation.**

(A) Mice were treated as described in Fig. 1G. mRNA expression of *TNF-α*, *IL-1β*, *IL-6* and *IL-23* in colons were detected using qPCR. (B) Mice were treated as described in Fig. 1C. Quantification of the macrophages (CD11b^+^ F4/80^+^) or neutrophils (CD11b^+^ Gr-1^+^), TH1 (CD3^+^ CD4^+^ IFN-γ^+^), TH2 (CD3^+^ CD4^+^ IL-4^+^) or Treg (CD3^+^CD4^+^ CD25^+^ Foxp3^+^) was detected by flow cytometry in LP. (C-D) Mice were treated as described in Fig. 1G. (C) Quantification of DCs, macrophages or neutrophilsw were detected by flow cytometry in LP. (D) Quantification of the TH17, TH1, TH2 or Treg was detected by flow cytometry in LP*.* For (A)-(D), each dot represents an individual mouse. (E) Production of IFN-γ, IL-4 or TGF-β in cellular supernatant was assessed by ELISA. (F) The percentage of IFN-γ^+^, IL-4^+^ or CD25^+^Foxp3^+^ CD4^+^ T cells was detected by flow cytometry. Data with error bars are represented as mean±SD. Each panel is a representative experiment of at least three independent biological replicates.


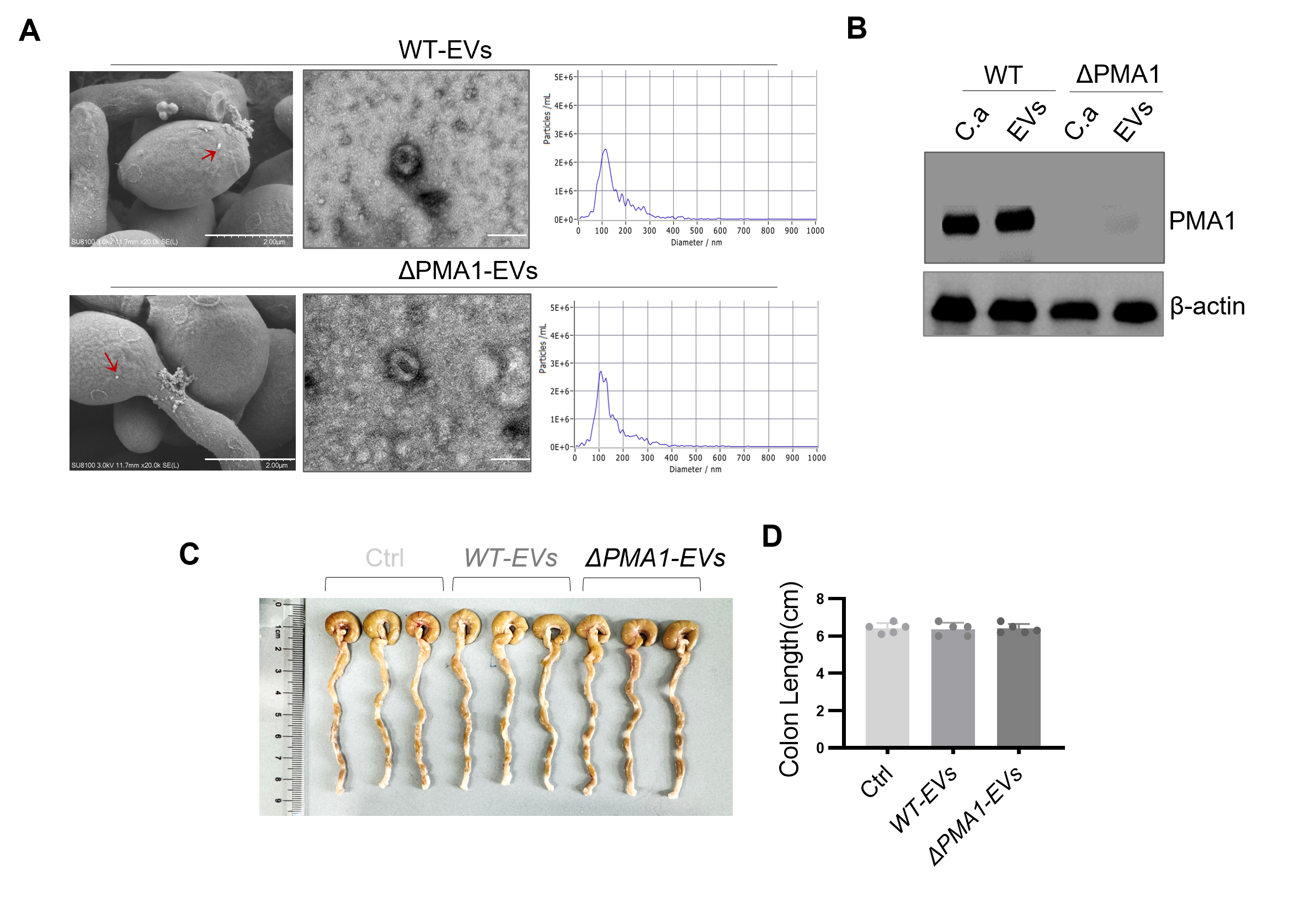


**Figure S3. *C. albicans* EVs containing PMA1 aggravate colitis.**

(A) SEM images of *WT-C.a* or *ΔPMA1-C.a* cells (left), TEM images of purified EVs obtained by ultracentrifugation of culture supernatants from *WT-C.a* or *ΔPMA1-C.a* (middle) and the particle size of EVs was analyzed by nanoflow (right). Scale bar represents 2μm. (B) Western blot analysis of PMA1 identified in *WT-C.a* or *ΔPMA1-C.a* and in both EVs samples. (C-D) Mice were gavaged every other day with PBS, WT-EVs, ΔPMA1-EVs (n=5 of each group). Colon length were measured, each dot represents an individual mouse.


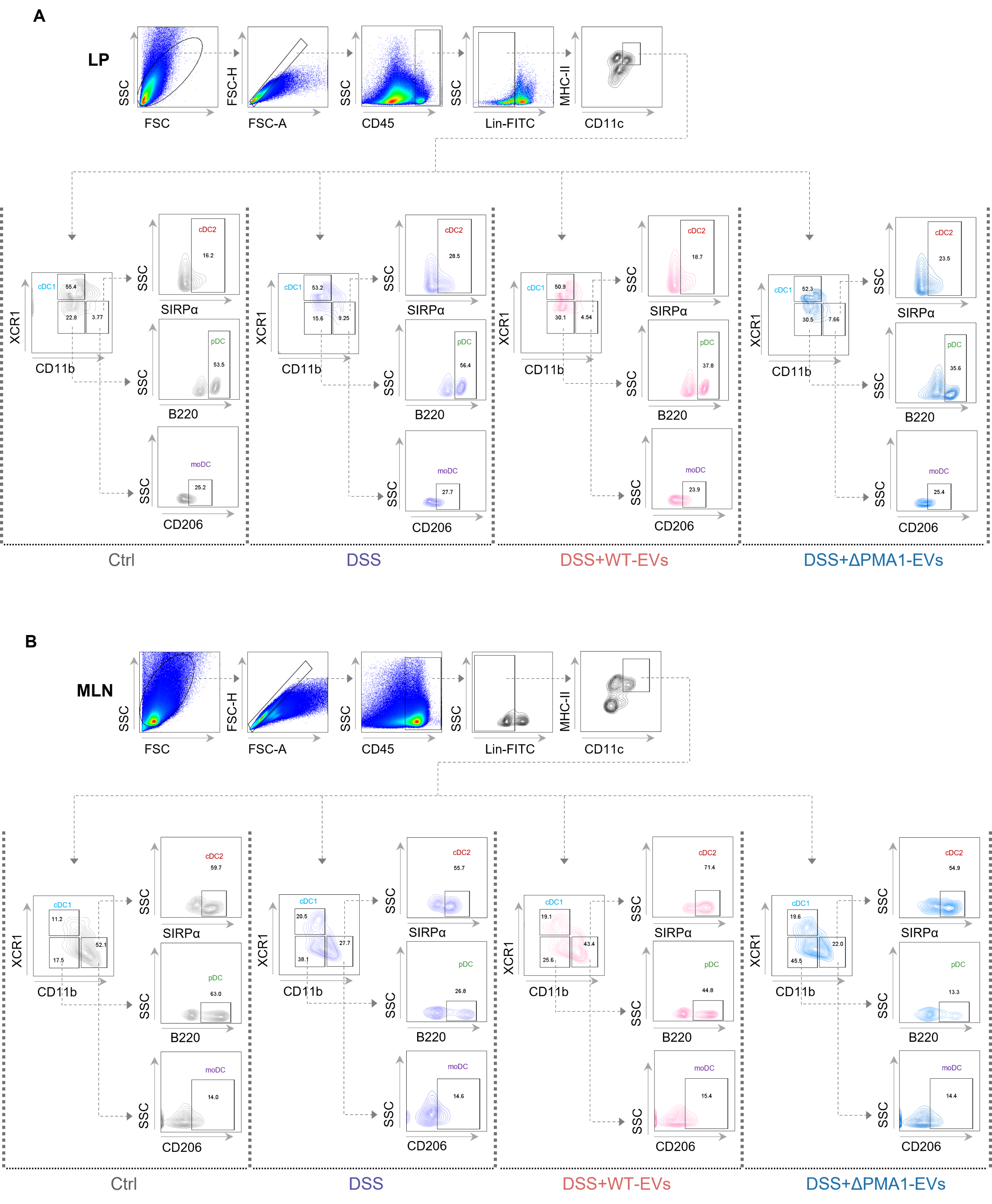


**Figure S4. The gating strategy of DC subsets in the gut.**

(A) The gating strategy of the cDC1 subset ((Lin:CD3, CD14, CD16, CD64)^-^ CD11c^+^ MHCII^+^ XCR1^+^CD11b^-^), the cDC2 subset ((Lin:CD3, CD14, CD16, CD64)^-^ CD11c^+^ MHCII^+^ XCR1^-^ CD11b^+^ SIRPα^+^), the pDC subset ((Lin:CD3, CD14, CD16, CD64)^-^ CD11c^+^ MHCII^+^ XCR1^-^ CD11b^-^ B220^+^) and the moDC subset ((Lin:CD3, CD14, CD16, CD64)^-^ CD11c^+^ MHCII^+^ XCR1^-^CD11b^+^ CD206^+^) in LP. (B) The gating strategy of the cDC1 subset ((Lin:CD3, CD14, CD16, CD64)- CD11c+ MHCII+ XCR1+CD11b-), the cDC2 subset ((Lin:CD3, CD14, CD16, CD64)- CD11c+ MHCII+ XCR1- CD11b+ SIRPα+), the pDC subset ((Lin:CD3, CD14, CD16, CD64)- CD11c+ MHCII+ XCR1- CD11b- B220+) and the moDC subset ((Lin:CD3, CD14, CD16, CD64)- CD11c+ MHCII+ XCR1-CD11b+ CD206+) in MLN.


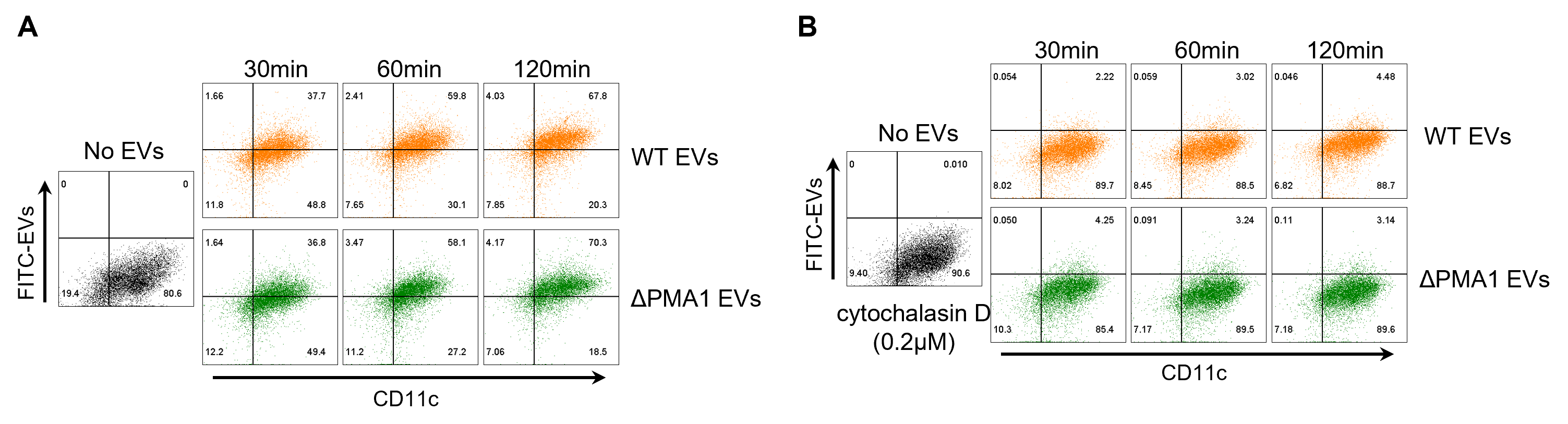


**Figure S5. Deletion of PMA1 in EVs did not affect its uptake by DCs and did not affect the immune response of macrophages.**

(A) BMDCs stimulated with FITC-labeled WT-EVs or ΔPMA1-EVs for 24 hours, FITC fluorescence were detected by flow cytometry. (B) BMDCs were pretreated with cytochalasin (0.2μm), and then stimulated with FITC-labeled WT-EVs or ΔPMA1-EVs for 24 hours. FITC fluorescence were detected by flow cytometry.


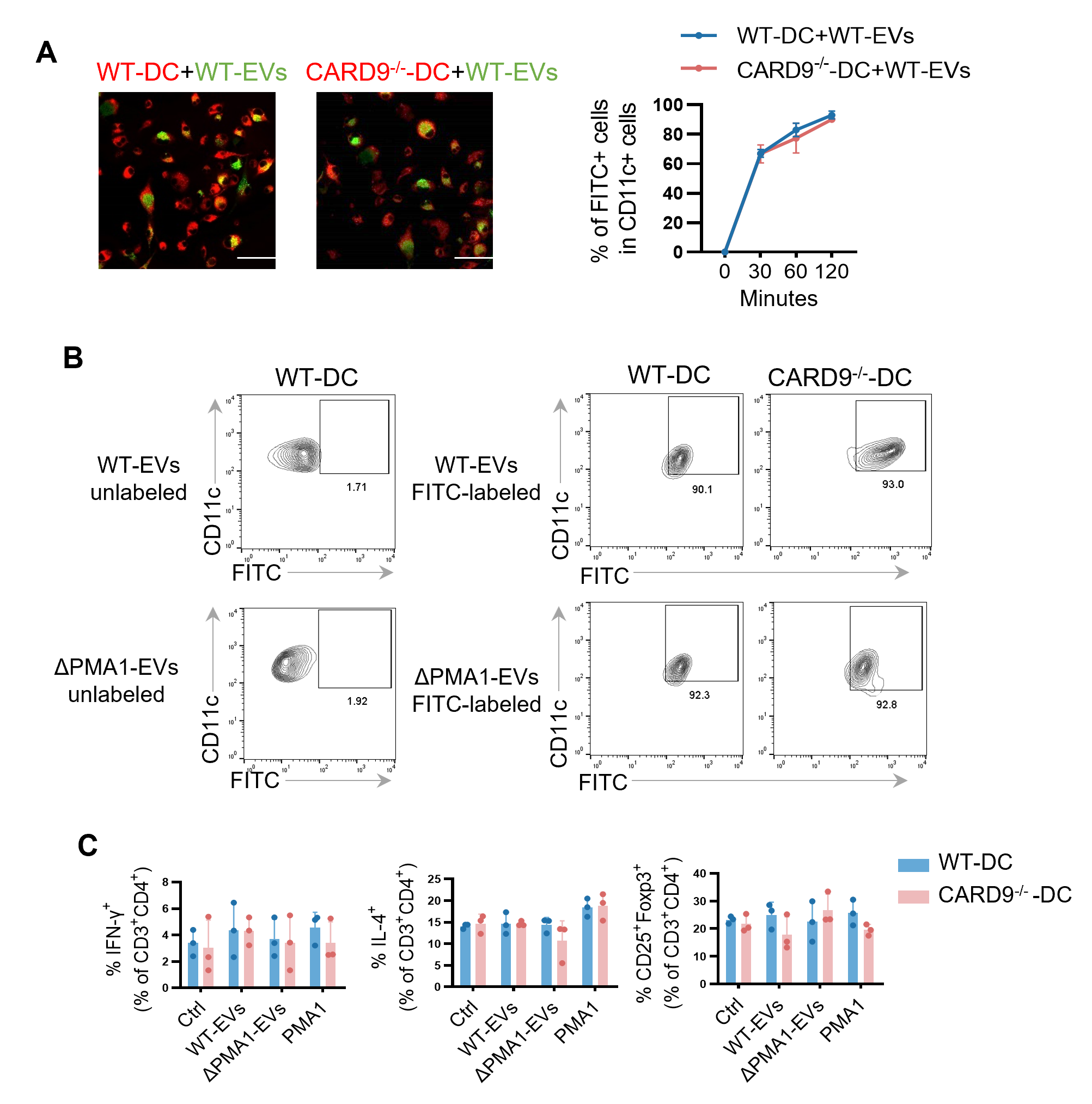


**Figure S6. CARD9 did not affect EVs uptake by DCs.**

(A) WT-EVs were labeled with FITC (green) and incubated with WT-DC or CARD9^-/-^-DC. Cells were fixed and cell membrane was stained with DiI (red), scale bar represents 20μm (left). WT-DC or CARD9^-/-^-DC were treated with FITC-labeled WT-EVs for 30, 60, 120 min, FITC fluorescence were detected by flow cytometry. (B) WT-DC or CARD9^-/-^-DC were treated with FITC-labeled WT-EVs or ΔPMA1-EVs for 120 min, FITC fluorescence were detected by flow cytometry. (D) The percentage of IFN-γ^+^, IL-4^+^ or CD25^+^Foxp3^+^ CD4^+^ T cells was detected by flow cytometry. Data with error bars are represented as mean±SD. Each panel is a representative experiment of at least three independent biological replicates.

**Table S1: Demographic results of controls and patients with ulcerative colitis**

| Clinical parameter | Control | UC |
| --- | --- | --- |
| Total n | 5 | 5 |
| Age | 44(27-67) | 46(27-71) |
| Male/Female | 2/3 | 2/3 |
| Active smoking (yes/no) | 0/5 | 0/5 |
| Flare/remission | N/A | 5/0 |
| Montreal classification (E1/E2 /E3) | N/A | 2/3/0 |
| Treatment: 5-ASA (yes/no) | N/A | 0/5 |
| Treatment: Corticosteroids (yes/no) | N/A | 0/5 |
| Treatment: Thiopurine or Methotrexate (yes/no) | N/A | 0/5 |
| Treatment: anti-TNF alpha (yes/no) | N/A | 0/5 |
| Treatment: Antibiotics (yes/no) | N/A | 0/5 |

“N/A” represents not applicable. If applicable, values are presented as median and range in brackets.

**Table S2: Primers used in this study**

| **Gene** | **Application** | **Primer** | **Sequence** |
| --- | --- | --- | --- |
| 18S rRNA | qPCR | F | 5'-GGRAAACTCACCAGGTCCAG -3' |
|  |  | R | 5'-GSWCTATCCCCAKCACGA - 3' |
| *C. albicans ITS* | qPCR | F | 5'-CTTTGACAATGGCTTAGGTCTAAC-3' |
|  |  | R | 5'-GTTGGTTTCTTTTCCTCC-3' |
| *C. albicans PMA1* | qPCR | F | 5'-TCTTCCTTCAAAGCCGTCCC-3' |
|  |  | R | 5'-GGCAAACCAACTGGAACACC-3' |
| Mouse *β-actin* | qPCR | F | 5'-GTGACGTTGACATCCGTAAAGA-3' |
|  |  | R | 5'-GCCGGACTCATCGTACTCC-3' |
| Mouse *TNF-α* | qPCR | F | 5'-CAGGCGGTGCCTATGTCTC-3' |
|  |  | R | 5'-CGATCACCCCGAAGTTCAGTAG-3' |
| Mouse *IL-6* | qPCR | F | 5'-CTGCAAGAGACTTCCATCCAG-3' |
|  |  | R | 5'-AGTGGTATAGACAGGTCTGTTGG-3' |
| Mouse *IL-23* | qPCR | F | 5'-CAGCAGCTCTCTCGGAATCTC-3' |
|  |  | R | 5'-TGGATACGGGGCACATTATTTTT-3' |
| Mouse *IL-17* | qPCR | F | 5'-TCAGCGTGTCCAAACACTGAG-3' |
|  |  | R | 5'-CGCCAAGGGAGTTAAAGACTT-3' |
| Mouse *IFN-γ* | qPCR | F | 5'-GCCACGGCACAGTCATTGA-3' |
|  |  | R | 5'-TGCTGATGGCCTGATTGTCTT -3' |
| Mouse *IL-4* | qPCR | F | 5'-GGTCTCAACCCCCAGCTAGT-3' |
|  |  | R | 5'-GCCGATGATCTCTCTCAAGTGAT-3' |
| Mouse *TGF-β* | qPCR | F | 5'-CCACCTGCAAGACCATCGAC-3' |
|  |  | R | 5'-CTGGCGAGCCTTAGTTTGGAC-3' |
| Mouse *ALDOA* | qPCR | F | 5'-AGTCCACCGGAAGCATTGC-3' |
|  |  | R | 5'-CAGCCCCTGGGTAGTTGTC-3' |
| Mouse *GAPDH* | qPCR | F | 5'-AGGTCGGTGTGAACGGATTTG-3' |
|  |  | R | 5'-GGGGTCGTTGATGGCAACA-3' |
| Mouse *PGK1* | qPCR | F | 5'-ATGTCGCTTTCCAACAAGCTG-3' |
|  |  | R | 5'-GCTCCATTGTCCAAGCAGAAT-3' |
| Mouse *CARD9* | qPCR | F | 5'-CACACCCTATCTACGCCAGTG-3' |
|  |  | R | 5'-GTGACTTTCCGGTATAACTGAGG-3' |
| Mouse *LDHA* | qPCR | F | 5'-CAAAGACTACTGTGTAACTGCGA-3' |
|  |  | R | 5'-TGGACTGTACTTGACAATGTTGG-3' |
| Mouse *IL-1β* | qPCR | F | 5'-GAAATGCCACCTTTTGACAGTG-3' |
|  |  | R | 5'-TGGATGCTCTCATCAGGACAG-3' |
